# Bidirectional Action of Cenicriviroc, a CCR2/CCR5 Antagonist, Results in Alleviation of Pain-Related Behaviors and Potentiation of Opioid Analgesia in Rats With Peripheral Neuropathy

**DOI:** 10.3389/fimmu.2020.615327

**Published:** 2020-12-21

**Authors:** Klaudia Kwiatkowski, Katarzyna Pawlik, Katarzyna Ciapała, Anna Piotrowska, Wioletta Makuch, Joanna Mika

**Affiliations:** Department of Pain Pharmacology, Maj Institute of Pharmacology, Polish Academy of Sciences, Krakow, Poland

**Keywords:** buprenorphine, CCL3, IL-1beta, IL-18, IL-6, microglia, morphine, opioid receptor

## Abstract

Clinical management of neuropathic pain is unsatisfactory, mainly due to its resistance to the effects of available analgesics, including opioids. Converging evidence indicates the functional interactions between chemokine and opioid receptors and their influence on nociceptive processes. Recent studies highlight that the CC chemokine receptors type 2 (CCR2) and 5 (CCR5) seem to be of particular interest. Therefore, in this study, we investigated the effects of the dual CCR2/CCR5 antagonist, cenicriviroc, on pain-related behaviors, neuroimmune processes, and the efficacy of opioids in rats after chronic constriction injury (CCI) of the sciatic nerve. To define the mechanisms of action of cenicriviroc, we studied changes in the activation/influx of glial and immune cells and, simultaneously, the expression level of *CCR2*, *CCR5*, and important pronociceptive cytokines in the spinal cord and dorsal root ganglia (DRG). We demonstrated that repeated intrathecal injections of cenicriviroc, in a dose-dependent manner, alleviated hypersensitivity to mechanical and thermal stimuli in rats after sciatic nerve injury, as measured by von Frey and cold plate tests. Behavioral effects were associated with the beneficial impact of cenicriviroc on the activation/influx level of C1q/IBA-1-positive cells in the spinal cord and/or DRG and GFAP-positive cells in DRG. In parallel, administration of cenicriviroc decreased the expression of CCR2 in the spinal cord and CCR5 in DRG. Concomitantly, we observed that the level of important pronociceptive factors (e.g., IL-1beta, IL-6, IL-18, and CCL3) were increased in the lumbar spinal cord and/or DRG 7 days following injury, and cenicriviroc was able to prevent these changes. Additionally, repeated administration of this dual CCR2/CCR5 antagonist enhanced the analgesic effects of morphine and buprenorphine in neuropathic rats, which can be associated with the ability of cenicriviroc to prevent nerve injury-induced downregulation of all opioid receptors at the DRG level. Overall, our results suggest that pharmacological modulation based on the simultaneous blockade of CCR2 and CCR5 may serve as an innovative strategy for the treatment of neuropathic pain, as well as in combination with opioids.

## Introduction

Neuropathic pain is a chronic condition resulting from damage to somatosensory neurons in the peripheral and central nervous system. Therapeutic management of patients with neuropathy remains extremely difficult due to the multifactorial pathogenesis and complex mechanisms involved in the generation and maintenance of painful symptoms (1). The interactions between neurons, glial, and immune cells are of a key importance for neuropathic pain development. Until now, it has been well established that activated non-neuronal cells are able to produce numerous factors, which are crucial for pathological nociceptive transmission after peripheral nerve injury (2–4). Glial and immune cells are known to express various receptors for cytokines, which may be a therapeutic target for novel drug development (2, 5). It has been previously demonstrated that intrathecal administration of IL-1beta (6), IL-6 (7), and some CC chemokine ligands (CCL), e.g., CCL2 (8), CCL3 (9), and CCL5 (10) induces neuropathic pain-related symptoms in healthy rodents. Thus, blocking the action or release of these molecules seems to be a promising direction for searching for new analgesics.

Recent studies highlight the importance of CC chemokine receptors type 2 and 5 (CCR2 and CCR5) in mediating pathological nociceptive processes (11–14). Both receptors belong to the CC subfamily, which is a category of integral membrane proteins representing G protein-coupled receptors (GPCRs). We have recently reported that selective blockade of CCR2 (14) and CCR5 (11, 12) induce analgesic effects in neuropathic rats, confirming their importance in nociception. Interestingly, it has been previously demonstrated that CCR2 and CCR5 may undergo heterodimerization, especially after co-stimulation of cells expressing both receptors with their ligands (15). The *in vitro* study revealed that these heterodimers are more efficient at inducing biological responses since they require lower chemokine concentrations for activity (16). This phenomenon seems to be an important issue, since under neuropathic pain conditions, we are dealing with uncontrolled activation of microglial cells appearing in association with a significantly enhanced level of endogenous ligands of these two receptors (11, 14, 17). The additional mechanism for modulating chemokine receptor activity is crosstalk interactions with opioid receptors, resulting in heterologous desensitization. Hence, cross-desensitization of the opioid receptors by endogenous ligands of CCR2 and CCR5 seems to change the balance between analgesia and hyperalgesia (18), which could be one of the reasons for the insufficient effectiveness of opioids in neuropathic pain therapies. Based on results, it would be of interest to examine whether a novel and largely interesting direction of study would be to examine whether the simultaneous blockade of these two receptors, using a dual antagonist, provides greater analgesic effects alone and in combination with opioids used in the clinic. Conducting such experiments is extremely important since polypharmacotherapy may terminate the necessity for using high doses of particular drugs and, thus, may reduce dangerous adverse effects of opioids. Additionally, here we used a compound that is currently undergoing clinical trials for other health problems, which seems to be important from clinical aspects.

Considering all these facts, we were interested in investigating the behavioral and molecular effects of the dual CCR2/CCR5 antagonist, cenicriviroc, under neuropathic pain conditions. In this study, we examined the dose-dependent effects of the dual CCR2/CCR5 antagonist on pain-related behaviors in rats after chronic constriction injury (CCI) of the sciatic nerve. To define molecular mechanisms of cenicriviroc action, we studied changes in the activation/influx of glial and immune cells and, simultaneously, the expression of CCR2, CCR5, and important pronociceptive cytokines (IL-1beta, IL-6, IL-18, CCL2, CCL3, CCL5) in the spinal cord and DRG. Furthermore, we assessed the influence of repeated administration of cenicriviroc on opioid efficacy in neuropathy. We also measured changes in the expression of opioid receptors after dual CCR2/CCR5 antagonist treatment in neuropathic rats.

## Materials and Methods

### Animals and Ethical Statement

Male Wistar rats (250–300 g) were provided by Charles River Laboratories International, Inc. (Germany) and housed in cages lined with sawdust under a standard 12/12-h light/dark cycle (light on at 8.00 a.m.) with food and water available *ad libitum.* Animals were allowed to acclimate to the environment for approximately 5 min prior to behavioral tests. This study was conducted in accordance with the recommendations of the International Association for the Study of Pain (IASP) (19) and the National Institutes of Health (NIH) Guide for the Care and Use of Laboratory Animals and was approved by the II Local Ethics Committee (permission number 1277/2015) based at the Maj Institute of Pharmacology, Polish Academy of Sciences (Krakow, Poland). Care was taken to minimize the amount and suffering of animals according to the 3R rule.

### Surgical Procedures

#### Intrathecal Catheter Implantation

Rats were prepared for intrathecal (*i.t*.) injections by the insertion of catheters under sodium pentobarbital anesthesia (60 mg/kg, *i.p*.) according to the methods of Yaksh and Rudy (20). The catheters consisted of 13-cm-long polyethylene tubing (PE 10, INTRAMEDIC, Clay Adams, Becton Dickinson and Company, Rutherford, NJ, USA) and were flushed with 70% (v/v) ethanol and then, before insertion, with water for injection. They were carefully introduced through the atlanto-occipital membrane to the subarachnoid space at the rostral level of the spinal cord lumbar enlargement (L4–L6), flushed slowly with 10 μl of water for injection, and the tip was tightened. After catheter implantation, the rats were allowed to recover for a minimum of one week before further experiments and monitored for physical impairments. After the surgery, all rats were fed separately. Animals with visible motor deficits were excluded from further study.

#### Chronic Constriction Injury

CCI of the sciatic nerve in rats was performed under sodium pentobarbital anesthesia (60 mg/kg, *i.p*.) according to the procedure described by Bennett and Xie (21). First, an incision was created below the hipbone, and the *biceps femoris* and *gluteus superficialis* were separated. The right sciatic nerve was exposed, and four ligatures (4/0 silk) were tied loosely around the nerve with 1-mm spacing until a brief twitch in the respective hind limb was elicited. After the surgery, the rats developed long-lasting neuropathic pain symptoms, such as hypersensitivity to the mechanical and thermal stimuli.

In all experiments as a control group, we used naive animals, so completely untreated rats, because in our previous studies we have shown that there is no difference in nociceptive response and protein levels of important pronociceptive factors between naive and sham-operated rats (22). Also, our newly conducted research showed that there is no significant difference in the protein levels of IBA-1 and GFAP between naive and sham-operated rats (**Supp. 1**). In contrast, significant changes in IBA-1 (**SFig. 1A**) and GFAP (**SFig. 1B**) protein level were observed in the CCI-exposed rats compared to naive and sham groups.

### Behavioral Tests

#### Von Frey Test

Mechanical hypersensitivity was measured using an automatic von Frey apparatus (Dynamic Plantar Aesthesiometer Cat. No. 37400, Ugo Basile, Gemonio, Varese, Italy), as described previously (12, 13). Rats were placed in plastic cages with a wire net floor 5 min before the experiment and allowed to move freely in this enclosed area. Von Frey filaments were applied in increasing values (up to 26 g) to the midplantar surface of the hind paw, and measurements were obtained automatically. The paw-withdrawal reflex was recorded as the force at which the paw was withdrawn.

#### Cold Plate Test

Thermal hypersensitivity was estimated using a Cold/Hot Plate Analgesia Meter (Cat. No. 05044, Columbus Instruments, Ohio, USA), according to our previous studies (12, 13). Rats were placed on the cold plate, and the latency to lift the hind paw was recorded. The temperature of the cold plate was maintained at 5^0^C, and cut-off latency was set to 30 s. In all cases, the injured paw reacted first.

### Drug Administration

**Figure 1:**
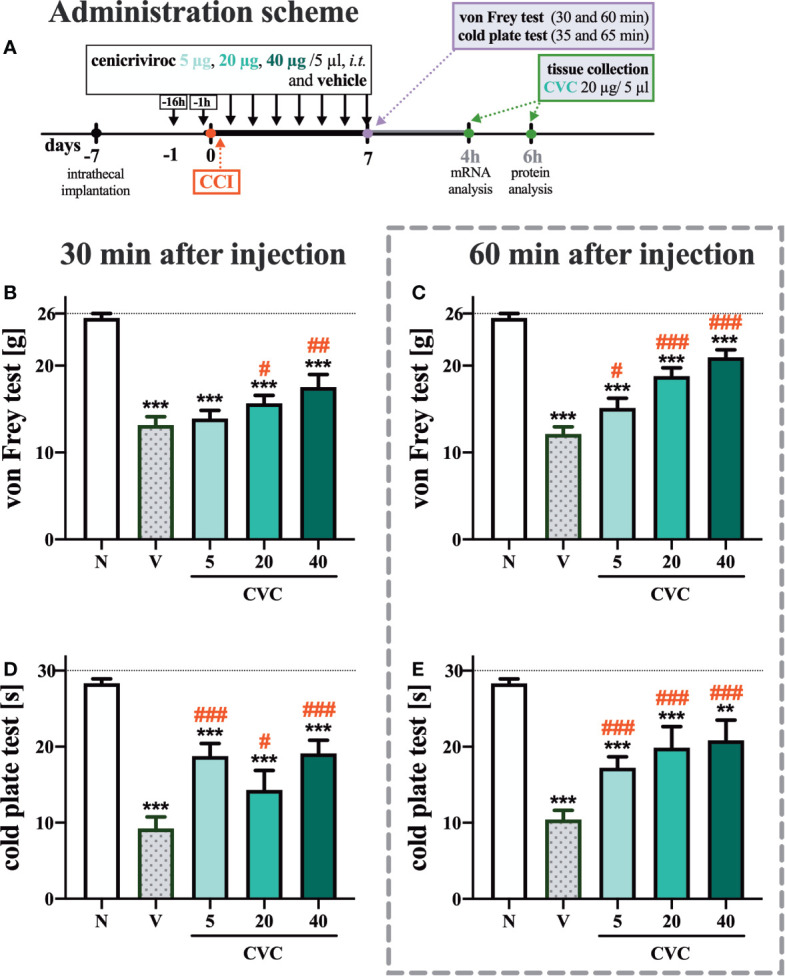
Dose-dependent changes following the preemptive (16 h and 1 h before CCI) and then repeated *i.t.* administration of cenicriviroc in concentrations of 5 µg, 20 µg, and 40 µg/5 µl [**(A)** administration scheme] on pain-related behaviors [**(B, C)** von Frey test; **(D, E)** cold plate test] on day 7 post CCI, 30 min or 60 min after cenicriviroc or vehicle injection. Data are presented as the mean ± SEM (n = 7–10 per group). Intergroup differences were analyzed by ANOVA with Bonferroni’s multiple comparisons test. ***p < 0.0001, **p < 0.01 indicates differences between naive and CCI-exposed rats; ^###^p < 0.001, ^##^p < 0.01, ^#^p < 0.05 indicates differences between V-treated and CVC-treated CCI-exposed rats. The dotted line shows the cut-off value. CCI, chronic constriction injury; CVC, cenicriviroc; N, naive; V, vehicle.

The following substances were used in the current experiments: cenicriviroc (**CVC**; Axon Medchem, Groningen, Netherlands), morphine hydrochloride (**M**; TEVA, Kutno, Poland), and buprenorphine (**B**; Polfa Warszawa S.A., Warsaw, Poland). Each compound was slowly delivered in a volume of 5 µl *via* the *i.t.* catheter, followed by 10 µl of water for injection. Cenicriviroc was dissolved in DMSO and administered *i.t.* preemptively 16 h and 1 h before CCI and then once a day for 7 days in concentrations of 5, 20, and 40 µg/5 µl. The control group received vehicle (V; DMSO) at the same schedule (**Figure 1A**). The behavioral tests were conducted 7 days after CCI always in the same order, first using the von Frey test and then the cold plate test at two time points, 30 min and 60 min after the last drug injection (**Figure 1A**). On day 7 post-CCI, chronically CVC-treated (20 µg/5 µl) and V-treated rats received a single dose of morphine or buprenorphine (2.5 µg/5 µl) 30 min after CVC/V injection, and then the behavioral tests were repeated (**Figure 6A**).

### Biochemical Analysis

#### RT-qPCR (Analysis of Gene Expression)

The ipsilateral sides of the dorsal lumbar (L4–L6) spinal cord and dorsal root ganglia (DRG; L4–L6) were collected immediately after decapitation 4 h after the last CVC administration on the 7^th^ day post-CCI. Total RNA was extracted using TRIzol reagent (Invitrogen, Carlsbad, California, USA) as described previously by Chomczynski and Sacchi (23). The RNA concentration was measured using a DeNovix DS-11 Spectrophotometer (DeNovix Inc., Wilmington, USA). Reverse transcription was performed using 1 μg of total RNA with Omniscript reverse transcriptase (Qiagen Inc., Hilden, Germany) at 37°C for 60 min. RT reactions were performed in the presence of an Omniscript RT Kit (Qiagen Inc., Hilden, Germany), RNAse inhibitor (rRNAsin, Promega, Mannheim, Germany) and an oligo (dT16) primer (Qiagen Inc., Hilden, Germany). The resulting cDNA was diluted 1:10 with H_2_O, and for each reaction, approximately 50 ng of cDNA synthesized from the total RNA of an individual animal was used for the quantitative real-time PCR (qRT-PCR) assay. The qRT-PCR was performed using Assay-On-Demand TaqMan probes according to the manufacturer’s protocol (Applied Biosystems, Foster City, CA, USA), and the reactions were performed on an iCycler device (Bio-Rad, Hercules, Warsaw, Poland). The following TaqMan primers and probes were used: Rn01527840_m1 (hypoxanthine-guanine phosphoribosyltransferase, *HPRT*); Rn01637698_s1 (*CC chemokine receptor type 2*; *CCR2*); Rn02132969_s1 (*CC chemokine receptor type 5*; *CCR5*); Rn00693288_m1 (*complement component 1q*; *C1q*); Rn00566603_m1 (*glial fibrillary acidic protein*; *GFAP*); Rn00562286_m1 (*CD4*); Rn00580577_m1 (*CD8*); Rn00580432_m1 (*interleukin 1 beta*; *IL-1beta*); Rn01410330_m1 (*interleukin 6*; *IL-6*); Rn01422083_m1 (*interleukin 18*; *IL-18*); Rn014566716_g1 (*CC chemokine ligand 2*; *CCL2*); Rn00564660_m1 (*CC chemokine ligand 3*; *CCL3*); Rn00579590_m1 (*CC chemokine ligand 5*; *CCL5*); Rn01430371_m1 (*OPRM*; *mu opioid receptor*; *MOR*); Rn00561699_m1 (*OPRD*; *delta opioid receptor*; *DOR*); Rn00567737_m1 (*OPRK*; *kappa opioid receptor*; *KOR*); Rn00440563_m1 (*OPRL*; *nociceptin receptor*; *NOR*). The amplification efficiency for each assay (between 1.7 and 2) was determined by running a standard dilution curve. The cycle threshold values were calculated automatically using CFX Manager v.2.1 software according to default parameters. The RNA abundance was calculated as 2^−(threshold cycle)^. Since the *HPRT* transcript level was not significantly changed among groups, we used it as an adequate housekeeping gene.

#### Western Blot (Analysis of Protein Levels)

The ipsilateral sides of the dorsal lumbar (L4–L6) spinal cord and DRG were collected immediately after decapitation 6 h after the last CVC administration on the 7^th^ day post-CCI, and then homogenized in RIPA buffer containing a protease inhibitor cocktail (Sigma-Aldrich, St. Louis, USA) and cleared *via* centrifugation (30 min, 14,000 rpm, 4°C). Total protein concentrations were measured using the bicinchoninic acid (BCA) method. Samples (10 μg of protein for spinal cord and 20 μg for DRG) were heated in loading buffer (4× Laemmli buffer, Bio-Rad, Warsaw, Poland) for 8 min at 98°C. Electrophoresis was performed on 4%–15% Criterion™ TGX™ pre-cast polyacrylamide gels (Bio-Rad, Warsaw, Poland). The proteins from the gels were transferred (semidry transfer, 30 min, 25 V) to Immun-Blot PVDF membranes (Bio-Rad, Warsaw, Poland), and the membranes were blocked for 1 h at room temperature using 5% nonfat dry milk (Bio-Rad, Warsaw, Poland) in Tris-buffered saline with 0.1% Tween-20 (TBST). Next, the membranes were washed in TBST buffer and incubated overnight at 4°C with the following primary antibodies: rabbit anti-IBA-1 (1:500, Novus, Abingdon, UK), rabbit anti-GFAP (1:10,000, Novus, Abingdon, UK), mouse anti-CD4 (1:1,000, R&D Systems, Minneapolis, MN), rabbit anti-CD8 (1:500, Santa Cruz, Dallas, TX), rabbit anti-IL-1beta (1:500, Abcam, Cambridge, UK), rabbit anti-IL-6 (1:500, Invitrogen, Carlsbad, CA), rabbit anti-IL-18 (1:500, Abcam, Cambridge, UK), and mouse anti-GAPDH (1:5,000, Millipore, Darmstadt, Germany); then, the membranes were washed in TBST buffer and incubated for 1 h at room temperature with HRP-conjugated secondary antibodies (Vector Laboratories, Burlingame, USA) at a dilution of 1:5,000. To dilute the primary and secondary antibodies, SignalBoost™ Immunoreaction Enhancer Kit (Millipore, Darmstadt, Germany) solution was used. Detection of selected proteins was performed using Clarity™ Western ECL Substrate (Bio-Rad, Warsaw, Poland) and visualized with a Fujifilm LAS-4000 FluorImager system. To quantify the relative levels of immunoreactivity, Fujifilm Multi Gauge software was used. The membranes for each Western blot analysis are presented in the **Supplementary Materials** (**Supp. 2**).

#### MILLIPLEX^®^ Multiplex Assays Using Luminex^®^ (Analysis of Protein Levels)

Tissue samples from the dorsal lumbar (L4–L6) spinal cord and DRG were collected and prepared for analysis in the same manner as described in *Western Blot (Analysis of Protein Levels)*. The protein concentrations of CCL2, CCL3, and CCL5 were determined in tissue homogenates 7 days post-operatively using a MILLIPLEX^®^ MAP Rat Cytokine Chemokine Magnetic Bead Panel Immunology Multiplex Assay (Merck Millipore, Burlington, Massachusetts, USA), according to the manufacturer’s instructions.

### Statistical Analyses

The behavioral tests analyses are presented in grams and seconds as the mean ± standard error of the mean (SEM). One-way analysis of variance (ANOVA) was used to evaluate the experimental results. Differences between groups were analyzed with Bonferroni’s post-hoc test. The data obtained from biochemical analyses are presented as the fold change compared with naive rats on the ipsilateral side of the dorsal lumbar spinal cord and DRG. The biochemical analyses are presented as the mean ± SEM, which represents normalized averages. The intergroup differences were analyzed using ANOVA with Bonferroni’s multiple comparisons post-hoc test. All graphs and statistical analyses were performed using Prism 8 (GraphPad Software, San Diego, USA). P < 0.05 indicated significant differences between groups.

## Results

### The Dose-Dependent Effect of Repeated Administration of Cenicriviroc on Neuropathic Pain-Related Symptoms in Rats 7 Days After CCI

In the von Frey test, strong mechanical hypersensitivity was observed 7 days after CCI at both examined time points compared to naive animals (**Figures 1B, C**). One hour after *i.t.* administration of cenicriviroc, a significant analgesic effect was observed at all tested doses, and a dose-dependent trend was clearly noticeable (**Figure 1C**), while 30 min after drug administration, only the two highest doses (**Figure 1B**) induced analgesia in neuropathic rats.

Furthermore, at 7 days after sciatic nerve injury, strong thermal hypersensitivity was evoked, as measured by the cold plate test (**Figures 1D, E**). Significant attenuation of those painful effects was observed for all tested doses at both 30 min (**Figure 1D**) and 60 min (**Figure 1E**) after cenicriviroc administration.

### The Influence of Cenicriviroc on mRNA Levels of *CCR2* and *CCR5* in the Spinal Cord and DRG 7 Days After CCI

**Figure 2:**
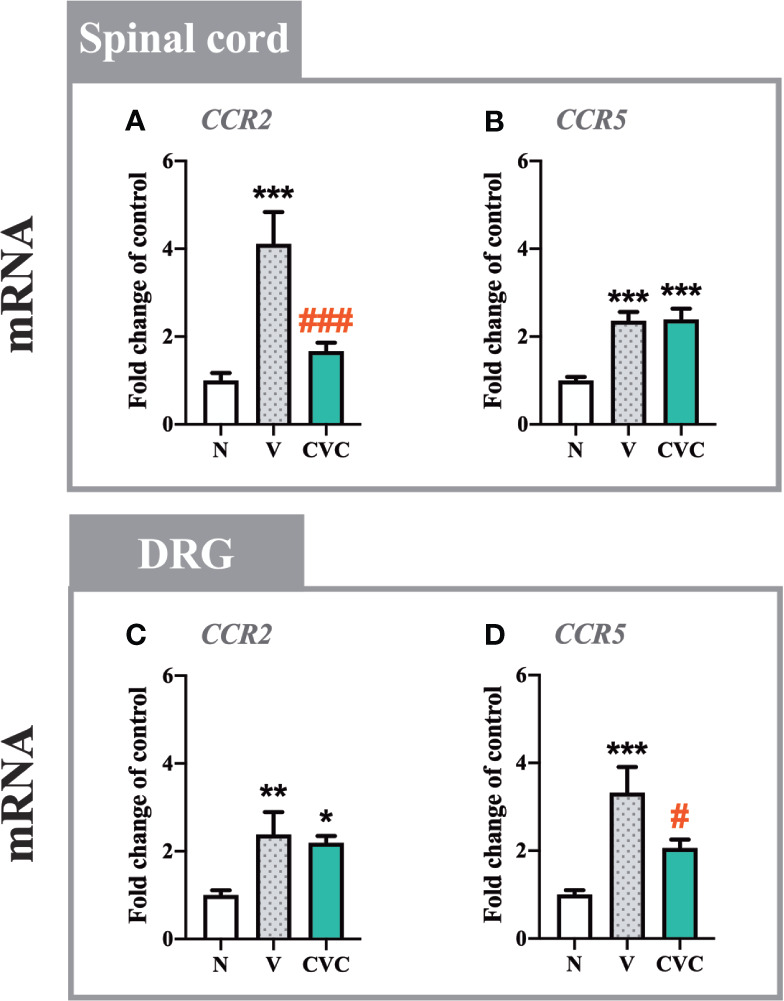
Changes in mRNA levels of *CCR2* and *CCR5* in the spinal cord **(A, B)** and DRG **(C, D)** on day 7 post CCI after repeated vehicle or cenicriviroc (20 µg/5 µl, *i.t.*) administrations, measured using RT-qPCR method. Data are presented as the mean ± SEM (n = 6–8 per group). Intergroup differences were analyzed by ANOVA with Bonferroni’s multiple comparisons test. ***p < 0.001, **p < 0.01, *p < 0.05 indicate differences between naive and V-treated/CVC-treated CCI-exposed rats; ^###^p < 0.001, ^#^p < 0.05 indicate differences between V-treated and CVC-treated CCI-exposed rats. CCI, chronic constriction injury; CVC, cenicriviroc; N, naive; V, vehicle.

In the spinal cord, a strong upregulation of *CCR2* and *CCR5* mRNA levels (**Figures 2A, B**) was observed 7 days after CCI. Cenicriviroc significantly prevented the upregulation of *CCR2* [F_(2,22)_ = 11.02, P = 0.0005, **Figure 2A**], while it did not affect *CCR5*.

In the DRG, both receptors were upregulated after nerve injury compared with naive animals (**Figures 2C, D**). In contrast to the spinal cord, the dual CCR2/CCR5 antagonist downregulated only the level of *CCR5* [F_(2,20)_ = 10.10, P = 0.0009, **Figure 2D**], but not *CCR2*.

### The Influence of Cenicriviroc on the mRNA and Protein Levels of *C1q*/IBA-1, GFAP, CD4, and CD8 in the Spinal Cord and DRG 7 Days After CCI

**Figure 3:**
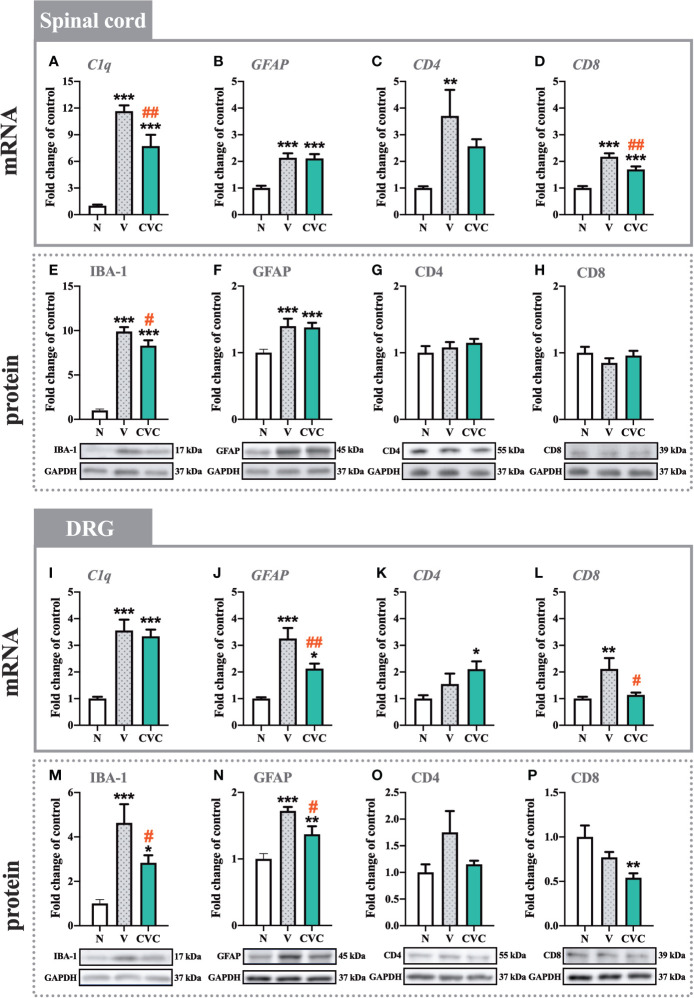
Changes in mRNA **(A–D, I–L)** and protein **(E–H, M–P)** levels of *C1q*/IBA-1, GFAP, CD4, and CD8 in the spinal cord **(A–H)** and DRG **(I–P)** on day 7 post CCI, after repeated vehicle or cenicriviroc (20 µg/5 µl, *i.t.*) administration, measured using RT-qPCR and Western blot method. Data are presented as the mean ± SEM (n = 4–8 per group for mRNA analysis and n = 5–9 per group for protein analysis). Intergroup differences were analyzed by ANOVA with Bonferroni’s multiple comparisons test. ***p < 0.001, **p < 0.01, and *p < 0.05 indicate differences between naive and V-treated/CVC-treated CCI-exposed rats; ^##^p < 0.01, ^#^p < 0.05 indicate differences between V-treated and CVC-treated CCI-exposed rats. CCI, chronic constriction injury; CVC, cenicriviroc; N, naive; V, vehicle.

In the spinal cord, a strong upregulation of *C1q* and IBA-1 levels was observed 7 days after CCI compared with naive rats (**Figures 3A, E**). Cenicriviroc significantly downregulated both mRNA [F_(2,21)_ = 42.25, P < 0.0001, **Figure 3A**] and protein [F_(2,16)_ = 121.9, P < 0.0001, **Figure 3E**] levels of microglia/macrophage markers. Moreover, injury of the sciatic nerve caused an increase in GFAP mRNA and protein levels (**Figures 3B, F**), but *i.t.* administration of the dual CCR2/CCR5 antagonist did not influence these levels. The mRNA expression of *CD4* was strongly enhanced after CCI, but no changes between vehicle- and cenicriviroc-treated animals were observed (**Figure 3C**). CCI did not influence the CD4 protein level (**Figure 3G**). Similarly, the protein level of CD8 was unchanged 7 days after CCI (**Figure 3H**), while its mRNA was strongly upregulated post injury, which was significantly decreased in response to cenicriviroc [F_(2,11)_ = 31.39, P < 0.0001, **Figure 3D**].

In the DRG, the mRNA level of *C1q* was upregulated after injury (**Figure 3I**); however, the dual antagonist did not impact these changes. We have observed an increase in IBA-1 protein level after CCI, and cenicriviroc effectively reduced these changes [F_(2,19)_ = 10.23, P = 0.0010, **Figure 3M**]. Additionally, strong increases in GFAP mRNA and protein levels were obtained after nerve injury, and in both cases, the CCR2/CCR5 antagonist prevented these changes [F_(2,19)_ = 17.57, P < 0.0001, **Figure 3J**; F_(2,14)_ = 18.52, P = 0.0001, **Figure 3N**]. The mRNA and protein level of CD4 remained unchanged after CCI (**Figures 3K, O**). Administration of cenicriviroc increased the *CD4* mRNA level compared with naive animals (**Figure 3K**), but no changes were observed in its protein level after antagonist treatment (**Figure 3O**). CCI also evoked *CD8* mRNA level upregulation, and cenicriviroc treatment significantly decreased this level [F_(2,20)_ = 5.942, P = 0.0094, **Figure 3L**]. A downregulation between the antagonist-treated and naive animals was also observed for the CD8 protein level (**Figure 3P**).

### The Influence of Cenicriviroc on the mRNA and Protein Levels of IL-1beta, IL-6, and IL-18 in the Spinal Cord and DRG 7 Days After CCI

**Figure 4:**
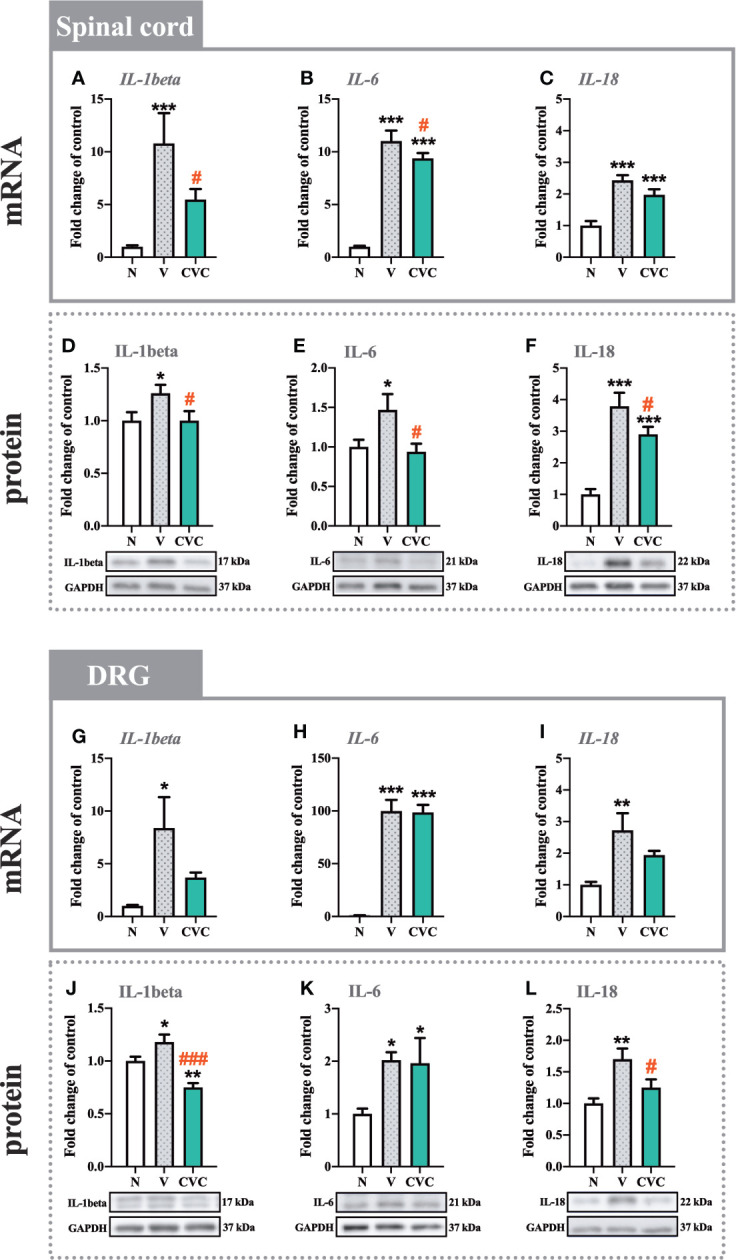
Changes in mRNA **(A–C, G–I)** and protein **(D–F, J–L)** levels of IL-1beta, IL-6 and IL-18 in the spinal cord **(A–F)** and DRG **(G–L)** on day 7 post CCI after repeated vehicle or cenicriviroc (20 µg/5 µl, *i.t.*) administration, measured using RT-qPCR and Western blot method. Data are presented as the mean ± SEM (n = 5–10 per group for mRNA analysis and n = 5-7 per group for protein analysis). Intergroup differences were analyzed by ANOVA with Bonferroni’s multiple comparisons test. ***p < 0.001, **p < 0.01, *p < 0.05 indicate differences between naive and V-treated/CVC-treated CCI-exposed rats; ^###^p < 0.001, ^#^p < 0.05 indicate differences between V-treated and CVC-treated CCI-exposed rats. CCI, chronic constriction injury; CVC, cenicriviroc; N, naive; V, vehicle.

In the spinal cord, the mRNA and protein levels of IL-1beta were significantly higher 7 days after CCI compared with naive animals (**Figures 4A, D**). Moreover, cenicriviroc strongly downregulated these changes [F_(2,21)_ = 7.793, P = 0.0029, **Figure 4A**; F_(2,19)_ = 3.406, P = 0.0544, **Figure 4D**]. A similar situation was obtained for IL-6, the mRNA and protein level of which increased after CCI (**Figures 4B, E**) and decreased after dual CCR2/CCR5 antagonist administration [F_(2,18)_ = 140.4, P < 0.0001, **Figure 4B**; F_(2,16)_ = 4.471, P = 0.0287, **Figure 4E**]. In addition, the protein level of IL-18 was enhanced after CCI. The antagonist significantly downregulated this level [F_(2,17)_ = 24.87, P < 0.0001, **Figure 4F**]. The mRNA expression of *IL-18* was also increased after sciatic nerve injury (**Figure 4C**), but no changes were observed after cenicriviroc administration.

In the DRG, strong mRNA upregulation was observed for *IL-1beta*, *IL-6* and *IL-18* (**Figures 4G–I**) after CCI compared with naive rats. For all the tested interleukins, no changes were observed after treatment with CCR2/CCR5 antagonist. In the case of the protein level, significant enhancement was also obtained for IL-1beta, IL-6 and IL-18 (**Figures 4J–L**). Cenicriviroc downregulated IL-1beta [F_(2,15)_ = 15.71, P = 0.0002, **Figure 4J**] and IL-18 [F_(2,10)_ = 6.660, P = 0.0145, **Figure 4L**] levels, while it had no impact on IL-6.

### The Influence of Cenicriviroc on the mRNA and Protein Levels of CCL2, CCL3, and CCL5 in the Spinal Cord and DRG 7 Days After CCI

**Figure 5:**
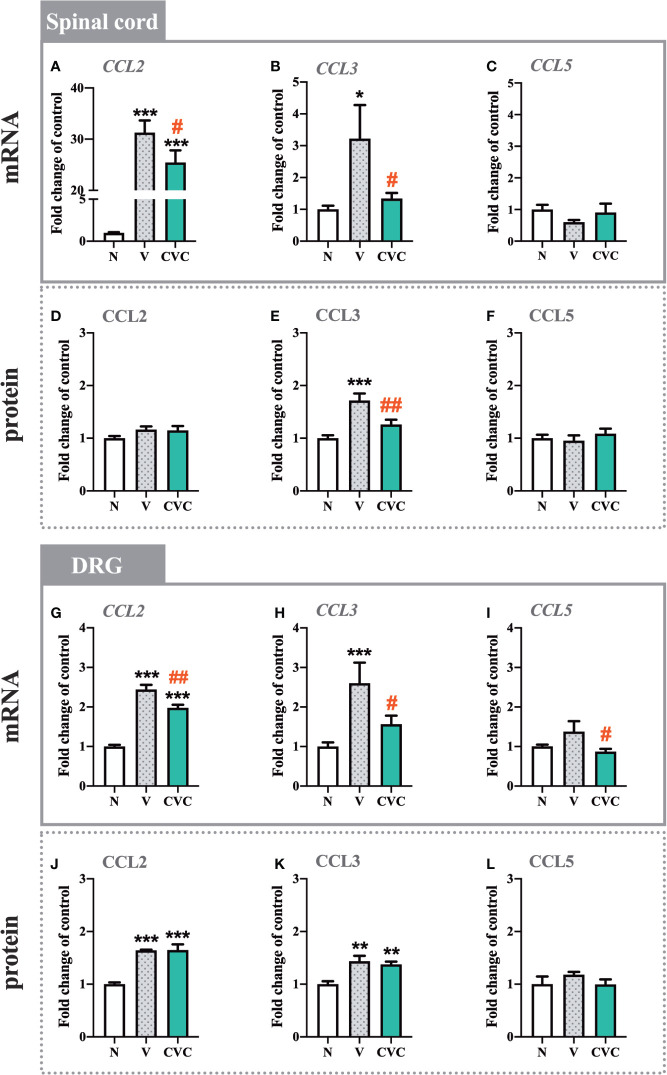
Changes in mRNA **(A–C, G–I)** and protein **(D–F, J–L)** levels of CCL2, CCL3 and CCL5 in the spinal cord **(A–F)** and DRG **(G–L)** on day 7 post CCI after repeated vehicle or cenicriviroc (20 µg/5 µl, *i.t.*) administration, measured using RT-qPCR and Luminex Assays. Data are presented as the mean ± SEM (n = 5**–**8 per group for mRNA analysis and n = 5**–**7 per group for protein analysis). Intergroup differences were analyzed by ANOVA with Bonferroni’s multiple comparisons test. ***p < 0.001, **p < 0.01, *p < 0.05 indicate differences between naive and V-treated/CVC-treated CCI-exposed rats; ^##^p < 0.01, ^#^p < 0.05 indicate differences between V-treated and CVC-treated CCI-exposed rats. CCI, chronic constriction injury; CVC, cenicriviroc; N, naive; V, vehicle.

The *CCL2* mRNA level was strongly upregulated in the spinal cord 7 days after CCI compared with naive rats. Cenicriviroc significantly prevented this change [F_(2,17)_ = 72.96, P < 0.0001, **Figure 5A**]. However, we did not observe any changes in the protein level of CCL2 (**Figure 5D**). Moreover, post-injury, spinally elevated *CCL3 mRNA* and protein levels were also downregulated by the CCR2/CCR5 antagonist [F_(2,16)_ = 3.998, P = 0.0391, **Figure 5B**; F_(2,15)_ = 13.85, P = 0.0004, **Figure 5E**]. Neither CCI nor drug administration impacted *CCL5* mRNA and protein levels in the spinal cord (**Figures 5C, F**).

In the DRG, the mRNA and protein levels of *CCL2* were enhanced after CCI compared with naive rats (**Figures 5G, J**). Administration of the dual antagonist prevented the mRNA increase in the DRG [F_(2,20)_ = 76.13, P < 0.0001, **Figure 5G**]. Similarly, the *CCL3* mRNA and protein level was significantly higher after sciatic nerve injury (**Figures 5H, K**). Cenicriviroc prevented its upregulation [F_(2,17)_ = 8.411, P = 0.0029, **Figure 5H**]. We did not observe changes in the mRNA and protein level of CCL5 after CCI (**Figures 5I, L**).

### The Influence of Cenicriviroc on the Analgesic Potency of Morphine and Buprenorphine 7 Days After CCI

**Figure 6:**
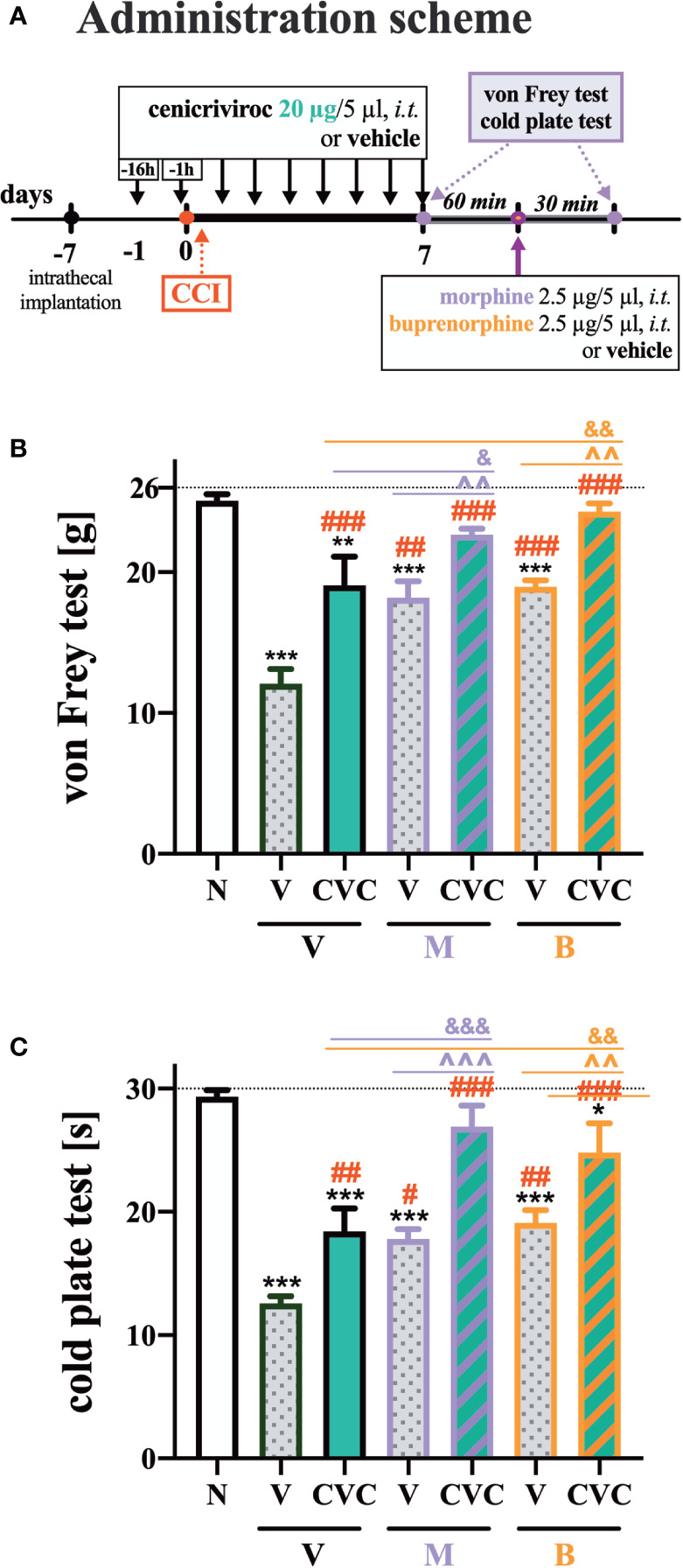
The influence of preemptive (16 h and 1 h before CCI) and then repeated *i.t.* administration of cenicriviroc in concentration of 20 µg/5 µl [**(A)** administration scheme] on opioid effectiveness, as measured by the von Frey **(B)** and cold plate **(C)** test at 7 days post CCI. On day 7, 60 min after the last cenicriviroc (**CVC**, 20 µg/5 µl) or vehicle (**V**) injection, rats received a single *i.t.* dose of morphine 2.5 µg/5 µl or buprenorphine 2.5 µg/5 µl. Behavioral tests were performed 30 min after opioid administration. The data are presented as the mean ± SEM (n = 6 per group). Intergroup differences were analyzed by ANOVA with Bonferroni’s multiple comparisons test. ***p < 0.001, **p < 0.01, *p < 0.05 indicate differences between naive and CCI-exposed rats; ^###^p < 0.001, ^##^p < 0.01, ^#^p < 0.05 indicate differences versus V + V-treated CCI-exposed rats; ^&&&^p < 0.001, ^&&^p < 0.01, ^&^p < 0.05 indicate differences between CVC + V- and CVC+ M/CVC+ B-treated, CCI-exposed rats; ^^^p < 0.001, ^^p < 0.01 indicate differences between V + M/V + B-treated and CVC + M/CVC + B-treated, CCI-exposed rats. The dotted line shows the cut-off value. B, buprenorphine; CCI, chronic constriction injury; CVC, cenicriviroc; M, morphine; N, naive; V, vehicle.

Repeated administration of cenicriviroc reduced pain‐like behaviors in both the von Frey and cold plate test (**Figures 6B, C**). Moreover, morphine and buprenorphine decreased mechanical hypersensitivity (**Figures 6B, C**) compared with vehicle-treated animals. The effectiveness of morphine and buprenorphine was enhanced in animals that were chronically treated with cenicriviroc, both in the von Frey (**Figure 6B**) and cold plate test (**Figure 6C**).

### The Influence of Cenicriviroc on the mRNA Levels of *MOR*, *DOR*, *KOR*, and *NOR* in the Spinal Cord and DRG 7 Days After CCI

**Figure 7:**
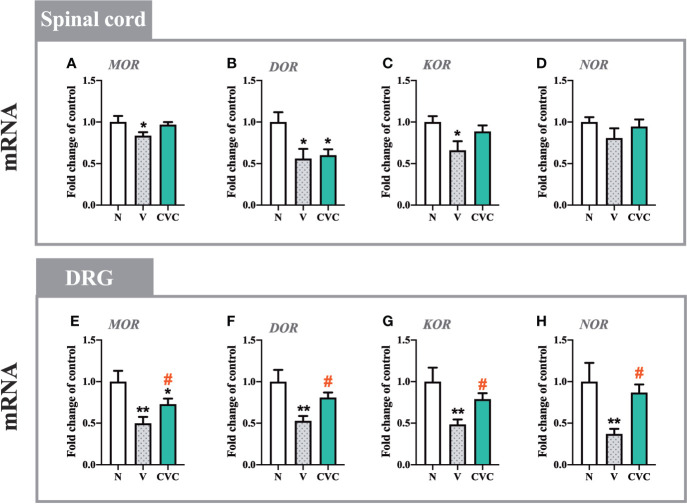
Changes in mRNA levels of *MOR*, *DOR*, *KOR* and *NOR* in the spinal cord **(A–D)** and DRG **(E–H)** on day 7 post CCI after repeated vehicle or cenicriviroc (20 µg/5 µl, *i.t.*) administration, measured using RT-qPCR method. Data are presented as the mean ± SEM (n = 4**–**8 per group). Intergroup differences were analyzed by ANOVA with Bonferroni’s multiple comparisons test. **p < 0.01, *p < 0.05 indicate differences between naive and V-treated/CVC-treated CCI-exposed rats; ^#^p < 0.05 indicates differences between V-treated and CVC-treated CCI-exposed rats. CCI, chronic constriction injury; CVC, cenicriviroc; N, naive; V, vehicle.

In the spinal cord, CCI led to the downregulation of mRNA levels of *MOR*, *DOR*, and *KOR*, but not *NOR* (**Figures 7A–D**). Cenicriviroc administration did not influence these receptors compared with vehicle-treated animals.

In the DRG, levels of *MOR*, *DOR*, *KOR and NOR* were significantly decreased after sciatic nerve injury (**Figures 7E–H**). The dual CCR2/CCR5 antagonist prevented the CCI-evoked downregulation of *MOR* [F_(2,16)_ = 7.437, P = 0.0052, **Figure 7E**], *DOR* [F_(2,18)_ = 7.483, P = 0.0043, **Figure 7F**], *KOR* [F_(2,19)_ = 6.705, P = 0.0063, **Figure 7G**], *NOR* [F_(2,20)_ = 5.035, P = 0.0169, **Figure 7H**].

## Discussion

Our behavioral studies demonstrated that repeated intrathecal injections of the dual CCR2/CCR5 antagonist cenicriviroc in a dose-dependent manner alleviated neuropathic pain-related behaviors in rats after sciatic nerve injury. Cenicriviroc decreased the activation and/or infiltration of IBA-1-positive cells (microglia/macrophages) in the spinal cord and DRG, and satellite cells in the DRG, and likely as a consequence reduced the level of some important pronociceptive factors (IL-1beta, IL-6, IL-18, and CCL3). Importantly, from a clinical perspective, cenicriviroc enhanced the analgesic potency of morphine and buprenorphine. These beneficial behavioral effects may result, among others, from the influence of cenicriviroc on the mRNA level of opioid receptors (MOR, DOR, KOR, and NOR) at the DRG level. Our results provide the first evidence that simultaneous targeting of CCR2 and CCR5 using cenicriviroc may have great potential for use in neuropathic pain therapies, especially since it is already under clinical trials, though in other health concerns.

The most recent studies have highlighted the importance of CCR2 and CCR5 in pathological nociceptive transmission under neuropathy (11–13, 24). Lee et al. (25) described a lower response to painful stimuli of CCR5-knockout than wild type mice. Similarly, impaired neuropathic pain responses in mice lacking CCR2 has been demonstrated (26). In our current studies, we demonstrated that *CCR2* and *CCR5* expression was increased in the spinal cord and DRG in rats after nerve injury, which is consistent with previously published data (2, 11, 13, 14, 27). Interestingly, cenicriviroc prevented nerve injury-induced upregulation of CCR2 in the spinal cord and CCR5 in the DRG. These chemokine receptors are expressed in both neuronal (28, 29) and non-neuronal (2, 26, 30–35) cells, such as microglia, astrocytes, macrophages, satellite cells, and infiltrating T lymphocytes. Here, we demonstrated that cenicriviroc might act as a microglia/macrophage and satellite cell activation inhibitor; in contrast, it did not influence the spinal level of the astrocyte activation. The time course of spinal gliosis occurring after peripheral nerve injury differs between microglia and astrocytes. Generally, microglial/macrophages activation is critical for the initial phase of neuropathic pain, and precedes and subsides before activation of astrocytes, which is rather related to the maintenance of neuropathic pain (36, 37). It was observed, that spinal microglia/macrophages react shortly after injury and reach maximal levels of activation in one week after nerve injury, and returns to baseline levels within 3 weeks. In contrast, spinal astrogliosis starts one week after injury, and persists for even several months (38). Cenicriviroc significantly reduced microglial/macrophages activation, but have no impact on astrogliosis. These results correlate well with previous data obtained after treatment with other chemokine receptors antagonist, e.g., CCR2 antagonist - RS504393 (13), CXCR3 antagonist - (±)-NBI-74330 (39), and CCR1 antagonist - J113863 (40). Recent data in the literature also indicate an important role of satellite glial cells in the development of painful neuropathy, thus also these cells became the subject of our research. Due to their unique location in DRG, these cells surround the cell bodies of sensory neurons and can strongly influence nociceptive transmission. The involvement of these cells after nerve injury is particularly related to the sudden increase in GFAP and the release of pronociceptive factors that stimulate the primary afferent endings in the dorsal horn spinal cord (41–43). Furthermore, T lymphocytes play an important role in the communication between the nervous and immune systems (44, 45). In DRG, T lymphocytes enhance the satellite cell response by synthesizing and releasing cytokines, including IL-1beta, IL-6, and CCL2, which directly modulate sensory neurons, leading to their hyperexcitability, through the activation of receptors located on their surface (44). These findings correlate well with our current studies, where we observed enhanced mRNA levels of CD4+ and CD8+ cell markers in the spinal cord and increased levels of CD8 in the DRG at 7 day after nerve injury. Cenicriviroc significantly decreased only the mRNA level of the CD8+ cell marker in the spinal cord and DRG, but this influence was not significant at the protein level as measured at day 7. Therefore, based on our studies conducted at this time point, it appears that cenicriviroc mainly benefited the activation of microglia/macrophages. Previous studies, including ours, have shown that minocycline, which acts as an inhibitor of microglia/macrophages, reduced hypersensitivity 7 days after nerve damage by lowering the level of pronociceptive factors, such as IL-1beta, IL-6, and IL-18 (46–50). Our previous results have also demonstrated that preemptive and repeated intrathecal injections of a selective CCR2 (RS504393) and CCR5 (maraviroc) antagonist attenuate neuropathic pain due to the inhibition of activation of IBA-1-positive cells (11, 14). In 2000, Wordliczek et al. reported in clinical investigations that patients who received preemptively a non-specific cytokine inhibitor, pentoxifylline, had lower opioid requirements in the early postoperative period, which was strongly associated with the lower serum levels of TNFα and IL-6 (51). Our research suggests that cenicriviroc might be effective in inhibiting the development of pain in the case of elective surgical interventions. Importantly, cenicriviroc appears to be particularly even more effective compound with analgesic properties, since, in contrast to selective antagonists, a single intrathecal and intraperitoneal injection can attenuate fully developed hypersensitivity to nociceptive stimuli in mice (17). Our current research sheds light on the molecular mechanisms underlying the beneficial long-lasting properties of chronic cenicriviroc treatment in neuropathic rats.

Based on our findings, we suggest that cenicriviroc may not only directly inhibit nociceptive transmission by blocking neuronal receptors but also inhibit, in direct and indirect manners, the excessive activation of microglial cells induced by peripheral nerve injury. Previous *in vitro* experiments have revealed that the majority of pronociceptive interleukins may originate from microglial cells, e.g., IL-1beta, IL-6, and IL-18 (52, 53). Furthermore, *ex vivo* experiments have demonstrated that in parallel to the activation of microglial cells, production of the abovementioned pronociceptive factors is increased (46, 48, 54–56). Released interleukins mediate central sensitization and, as a consequence, induce mechanical and thermal hypersensitivity (57). IL-1beta has been shown to play a crucial role in pathological changes that occur in different diseases with an immunological component, including neuropathic pain, which was also confirmed in our research (54, 58). Intrathecal injection of IL-1beta has been shown to evoke hypersensitivity in healthy rodents (59), and blockade of its receptor using an interleukin 1 receptor antagonist has provided pain relief in rats after sciatic nerve injury (54). Another pronociceptive factor belonging to the IL-1 superfamily is IL-18 (60, 61). Previous studies have demonstrated that nerve injury induces an increase in spinal mRNA and protein levels of IL-18 (55), which is consistent with our current results. Additionally, intrathecal administration of IL-18 causes behavioral and molecular changes similar to those observed after nerve injury (60). The cytokine IL-6 undergoes the strongest activation in many neuroimmune processes (62). Our biochemical experiments showed that cenicriviroc effectively prevented the enhanced increase in protein levels of IL-1beta, IL-6, and IL-18 in the spinal cord and/or DRG. Based on these results, we hypothesize that the strong impact of cenicriviroc on these interleukins is associated with the ability of that compound to inhibit microglia/macrophage activation and, as a result, the release of these pronociceptive interleukins.

Recent studies have highlighted that not only interleukins but also chemokines, play an important role in neuropathic pain pathogenesis. Importantly, in previous *in vitro* experiments, we demonstrated that CCR2 and CCR5 ligands, such as CCL2, CCL3, and CCL5, may originate from microglial cells (11, 13). The increasing number of studies indicate that neutralization of CCL2, acting *via* CCR2, might be effective in attenuating neuropathic pain (8) and cancer pain (63). The enhanced release of CCL2 by DRG neurons leads to the increased activation of spinal microglia (13, 64). The latest studies suggest that CCL2 produced by spinal neurons and astrocytes may also provoke a strong activation of microglial cells due to CCR2 located on their surface (3). Therefore, we suggest that the reduction of CCL2 activity related to the decrease in spinal CCR2 expression observed in cenicriviroc-treated rats is undoubtedly one explanation for the analgesic properties of this dual antagonist. Particularly, it is already known that CCL2 binding to CCR2 results in the phosphorylation of microglial p38MAPK, one of the main canonical signaling pathways in the nociceptive response. Activation of spinal microglia is critical in the pathogenesis of pain hypersensitivity following peripheral nerve injury (14, 24). Moreover, intrathecal injection of CCL2 induces long-lasting hypersensitivity and leads to strong activation of spinal microglial cells and production of pronociceptive cytokines (5, 24, 65). Other research groups have shown that mice overexpressing CCL2 exhibit increased hypersensitivity (66). Similarly, injection of CCL5, an endogenous ligand of CCR5, evokes neuropathic pain-like symptoms (67). Literature data show that CCL5 stimulates the influx of inflammatory cells and enhances the release of cytokines in injured nerves (10). In our study, we did not observe any changes in the level of CCL5 in the spinal cord and DRG after nerve injury; therefore, the analgesic properties of intrathecally administered cenicriviroc do not seem to result from the modulation of that chemokine activity in the examined structures. Another chemokine that acts *via* CCR5 and is presumably more important for nociception than previously thought is CCL3 (5). Changes in CCL3 levels have been observed in different neuropathic pain conditions (11, 28, 68, 69). Our biochemical analysis showed that after sciatic nerve injury, the mRNA and protein levels of CCL3 were significantly elevated in the spinal cord and DRG, which is consistent with others studies (11). A single intrathecal injection of CCL3 was reported to evoke strong pain-like behavior in naive mice, and direct neutralization of CCL3 reduces neuropathic pain in different animal models (28, 68, 70). Here, we demonstrated that cenicriviroc was able to effectively prevent the nerve injury-induced increase in CCL3 in the spinal cord and DRG, which appears to be an important aspect underlying the beneficial effects on the development of neuropathic pain observed in our studies, in particular with regard to the pleiotropic activity of CCL3.

A growing body of evidence suggests that modulation of neuroimmune interactions can potentiate opioid analgesic potency under neuropathic pain conditions. Opioids are commonly used in chronic pain therapies, but nevertheless, they are less effective in neuropathy than in other pain states (56, 71). The latest studies suggest a mutual connection between the immune and opioid system. Interestingly, neuroimmune mechanisms in neuropathic pain seem to be similar to those occurring in morphine tolerance development since non-neuronal cells become activated in both processes (56). The analgesic effects of opioids are modulated by activated microglia/macrophages releasing pronociceptive factors (e.g., IL-1beta and IL-18), which are able to reduce the opioid efficacy (46, 48, 72, 73). Thus, it has been suggested that the modulation of microglia/macrophage activity may lead not only to the attenuation of neuropathic pain but also to the improvement of morphine analgesic potency (74). In the current study, we demonstrated that the CCR2/CCR5 antagonist (cenicriviroc) injected repeatedly intrathecally diminished microglia/macrophage activation and, in parallel, enhanced the analgesic potency of opioids in neuropathic rats. Numerous studies have shown that the repeated administration of microglia/macrophage inhibitors, such as minocycline, delays the development of morphine tolerance due to the decrease in spinal level of IL-1beta and IL-18 (48, 72, 73). Therefore, it has been hypothesized that the excessive release of pronociceptive interleukins may modulate the activity of opioid receptors and, thus, decrease the analgesic potency of opioids observed in neuropathic pain pharmacotherapy. Importantly, cenicriviroc lowers the level of the above-mentioned interleukins, which may be one of the reasons for its beneficial influence on opioid efficacy. Furthermore, very recent research highlights the bidirectional regulation of the chemokine and opioid systems. Increased immunoreactivity of spinal CCL2 has been observed after chronic administrations of morphine. Moreover, neutralization of CCL2 prevents the development of morphine tolerance and diminishes spinal microglial activation (75). Our previous studies show that neutralization of CCL2 enhances the analgesic effects of morphine and buprenorphine in mice after nerve injury (8). CCL3 seems to have similar properties; however, to date, only one study has shown that treatment with neutralizing antibodies enhances morphine effectiveness (28). Our results reveal for the first time that cenicriviroc lowers the CCL3 level and simultaneously enhances opioid analgesia, suggesting its importance in opioid efficacy during neuropathic pain; however, further in-depth research is needed for confirmation. Additionally, our results provide the first evidence that cenicriviroc is able to enhance the analgesic potency of morphine and buprenorphine in rats with nerve injury, which is very important from a clinical perspective. The probable mechanisms underlying the beneficial effects of cenicriviroc on opioid efficacy are the inhibition of microglia/macrophage cell activation and, consequently, the lowering of spinal levels of crucial pronociceptive factors responsible for the suppression of opioid-induced analgesia, such as IL-1beta, IL-6, IL-18, and CCL3.

Among the mechanisms underlying the analgesic effects of the examined dual antagonist is its ability to simultaneously block two chemokine receptors important for nociceptive processes. They have been described as similar to opioid receptors belonging to the GPCRs, suggesting a potential cross-talk between them (18). One of the mechanisms governing the function of GPCRs is the heterologous desensitization that occurs when the activation of one type of a receptor leads to the suppressed activity of other types and regulation of the function of its ligands (76, 77). Recent studies have reported that the activation of CCR2 or CCR5 leads to the cross-desensitization of chemotactic activities of both MOR and DOR and that it is a bidirectional process, which has crucial implications for the immune response as well as nociceptive transmission (78). Based on heterologous desensitization, the neuronal signaling pathway involved in decreasing pain sensations, mediated by opioids, can be inactivated by chemokines (18). Lee et al. demonstrated that a deficiency of CCR5 is associated with MOR upregulation, with no increase in the desensitization of that receptor, and suggested that this phenomenon might be responsible for the increased analgesic effects against painful stimuli (25). In turn, the second mechanism is associated with the formation of heteromers. The discovery of MOR-CCR5 heterodimerization offers an interesting level of potential complexity in the negative cross-talk between opioid and chemokine receptors, during which the activation of one dimer partner leads to an inhibition in the function of the other partner (25, 78, 79). These reports are in strong association with our results concerning changes in CCR2 and CCR5 expression, where we demonstrated for the first time that chronic treatment of cenicriviroc effectively prevented their upregulation in the spinal cord and DRG, respectively, and simultaneously prevented the nerve injury-induced downregulation of all examined opioid receptors at the DRG level, as observed in our study and in agreement with others (80, 81). We assessed only the mRNA level of GPCRs, since associated changes in the protein level measured in overall tissue homogenate may not be accurate, because of the rapid internalization of these receptors upon agonist stimulation and subsequent degradation and/or transport to a recycling compartment to the cell surface for continued ligand binding. However, our study gave first evidence that cenicriviroc can beneficially influence transcription of opioid receptors in the DRGs. Based on citied literature we suggest, that the negative influence of opioid receptor activation on the functio of chemokine receptors may occur at the level of their conformation (82, 83). Therefore, the observed changes may have functional consequences, including an increase in the effectiveness of opioids, but the mechanisms underlying this phenomenon still require detailed explanation. The observed influence of the dual CCR2/CCR5 antagonist on the biosynthesis of chemokines and opioid receptors during neuropathic pain provides further confirmation of its beneficial mechanism of action.

Increasing evidence supports the excellent antinociceptive activity of bifunctional compounds in several animal models and indicates a significant advantage of compounds targeting more than one molecular target over the physical mixture of individual pharmacophores with respect to their analgesic effect (84–86). The benefits of this approach may arise from the simultaneous access to the two receptors at the same dose and, thus, the changing pharmacokinetics and pharmacodynamics, consequently leading to better analgesic effects. Furthermore, keeping in mind that cenicriviroc is undergoing analysis in a phase 2b clinical trial for the treatment of HIV-infected adults and has just entered the 3^rd^ phase of clinical trial for the treatment of liver fibrosis in adult subjects with non-alcoholic steatohepatitis, the big advantage of our studies is that such therapies would not incur high costs related to the design of new drugs. Thus, we hope that the results of this study will contribute to the creation of an effective and long-lasting therapy based on the modulation of two chemokine receptors, CCR2 and CCR5. Combination therapy, based on conventionally used opioids and antagonists of CCR2 and/or CCR5, seems promising and will hopefully yield satisfactory results.

## Conclusion

Cenicriviroc restores the neuroimmune balance by inhibiting the activation of macrophages/microglia and satellite cells, which in turn leads to a decrease in the level of important pronociceptive cytokines (IL-1beta, IL-6, IL-18, CCL3) in the spinal cord and/or DRG. Importantly, it also has a beneficial influence on the biosynthesis of CCR2, CCR5, and opioid receptors. The strong involvement of CCR2 and CCR5 in neuropathic pain may suggest that they may serve together as a potential therapeutic target, as well as in a combined therapy with opioids. However, further experimental and clinical research is needed to validate these hypotheses.

## Data Availability Statement

The original contributions presented in the study are included in the article/**Supplementary Material**. Further inquiries can be directed to the corresponding author.

## Ethics Statement

The animal study was reviewed and approved by II Local Ethics Committee Kraków Poland.

## Author Contributions

KK and JM planned the study. KK, KP, KC, AP, WM, and JM have made substantial contributions to the conception and design of the study, execution of the experiments, analysis and interpretation of data, and preparation of the manuscript. All authors provided final approval of the version to be published and agreed to be accountable for all aspects of the research in ensuring that questions related to the accuracy or integrity of any part of the study are appropriately investigated and resolved. All authors contributed to the article and approved the submitted version.

## Funding

This work was supported by the National Science Centre, Poland (PRELUDIUM 2018/29/N/NZ7/00287 and OPUS 11 2016/21/B/NZ4/00128) and by statutory funds of the Maj Institute of Pharmacology, Polish Academy of Sciences, Department of Pain Pharmacology. KK is funded by a scholarship START from Foundation for Polish Science. AP is a scholarship holder funded by the Ministry of Science and Higher Education, Poland. Maj Institute of Pharmacology Polish Academy of Sciences supported the open access publication.

## Conflict of Interest

The authors declare that the research was conducted in the absence of any commercial or financial relationships that could be construed as a potential conflict of interest.
