# Supplementary material for: Bidirectional Action of Cenicriviroc, a CCR2/CCR5 Antagonist, Results in Alleviation of Pain-Related Behaviors and Potentiation of Opioid Analgesia in Rats With Peripheral Neuropathy
Source: Front Immunol. 2020 Dec 21;11:615327. doi: 10.3389/fimmu.2020.615327 (PMC7779470; doi:10.3389/fimmu.2020.615327)
Supplement: Supplementary file 1 [file DataSheet_1.pdf]

SUPPLEMENTARY MATERIALS 1

The spinal changes in the protein level of IBA-1 and GFAP (microglia/macrophages and astrocytes activation markers, respectively) 7 days after chronic constriction injury (CCI) of the sciatic nerve in rats.

No significant difference between naive and sham-operated rats was observed in the spinal protein level for IBA-1 (Fig. SFig.1A) and GFAP (SFig.1B). In contrast, significant changes in the protein level for IBA-1 (SFig.1A) and GFAP (SFig.1B) were observed in the CCI-exposed rats compared to naive and sham groups.

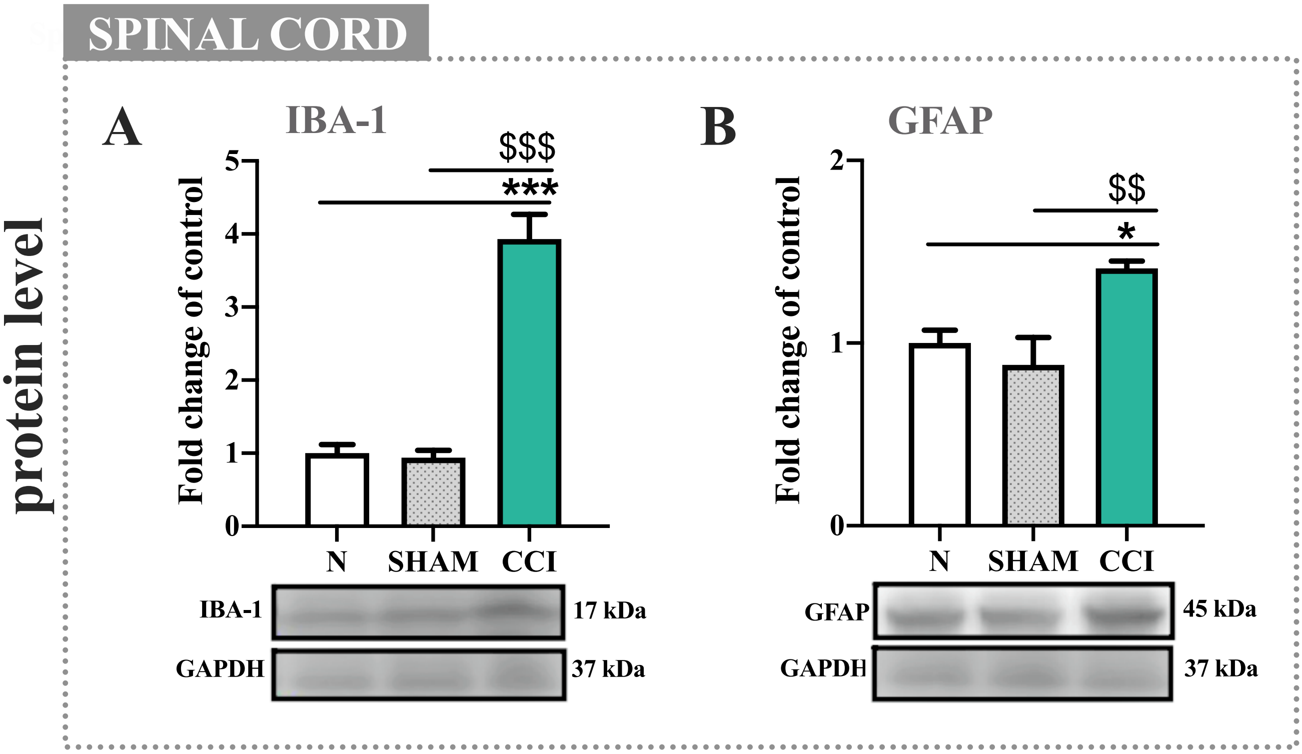

SFig 1. Changes in IBA-1 (**A**) and GFAP (**B**) protein levels in the spinal cord on the 7th day after CCI in rats. The data are presented as the mean fold changes with respect to the control ± SEM (5 samples per group). Intergroup differences were analyzed using ANOVA with Bonferroni's multiple comparisons test. *p<0.05, ***p<0.001 indicate difference versus naive rats; $$p<0.01, $$$p<0.001 indicate difference between sham-operated and CCI-exposed rats. *Abbreviations: CCI, operated animals with chronic constriction injury of the sciatic nerve; N, naive; SHAM, operated animals without chronic constriction injury of the sciatic nerve.*
