# Supplementary material for: Bidirectional Action of Cenicriviroc, a CCR2/CCR5 Antagonist, Results in Alleviation of Pain-Related Behaviors and Potentiation of Opioid Analgesia in Rats With Peripheral Neuropathy
Source: Front Immunol. 2020 Dec 21;11:615327. doi: 10.3389/fimmu.2020.615327 (PMC7779470; doi:10.3389/fimmu.2020.615327)

SPINAL CORD

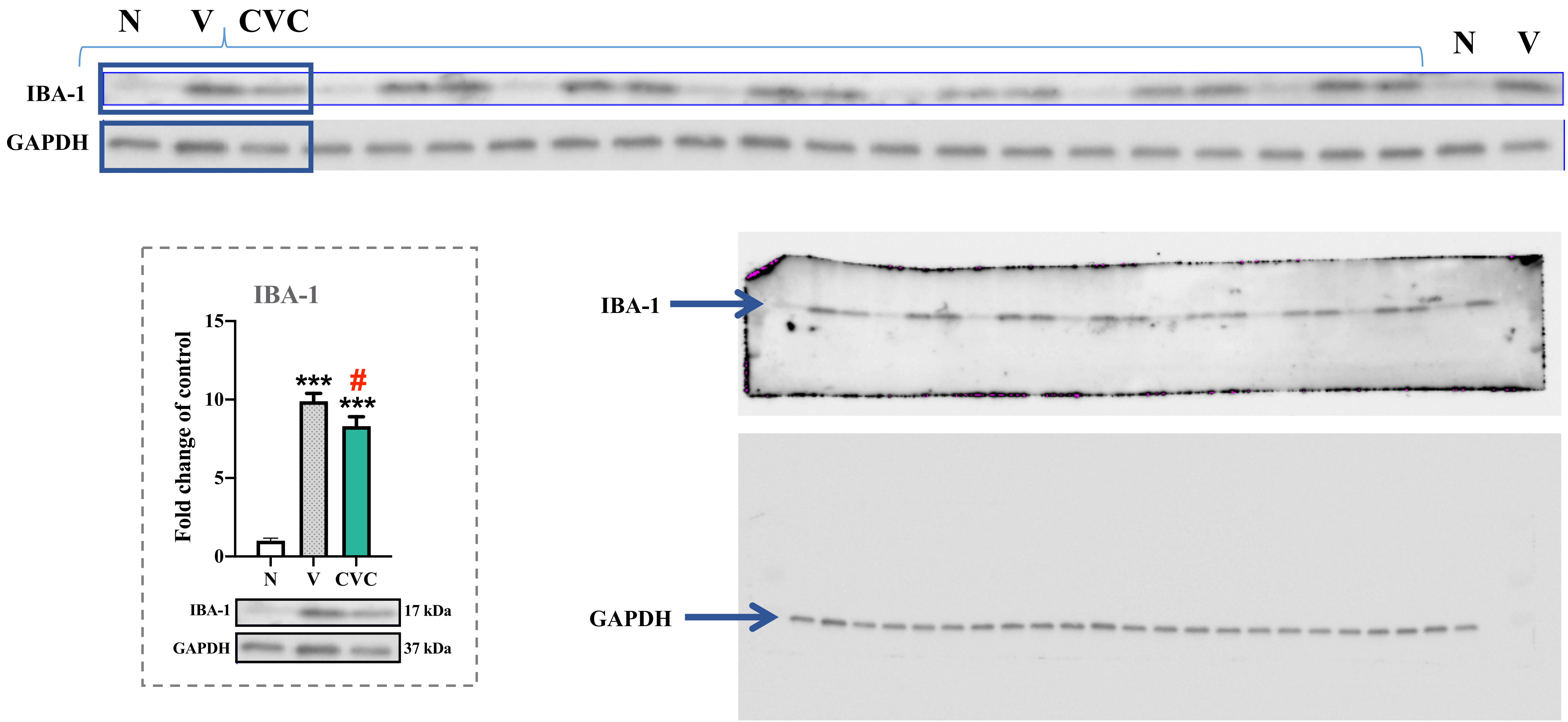
N
V
CVC
N
V
IBA-1
GAPDH
IBA-1
15
10
5
0
Fold change of control
***
***
#
N
V
CVC
IBA-1
17 kDa
GAPDH
37 kDa
IBA-1
GAPDH

SPINAL CORD

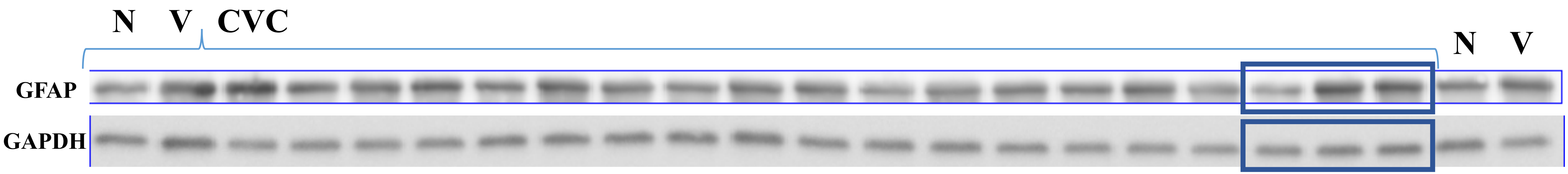

N V CVC
N V
GFAP
GAPDH

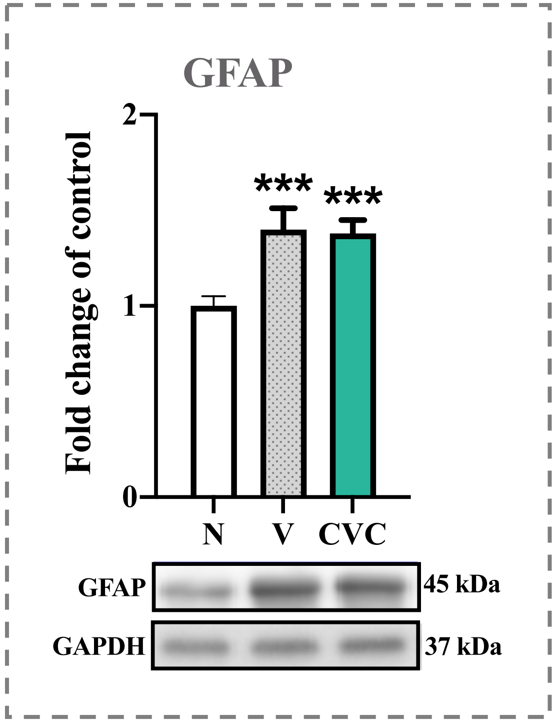

GFAP
Fold change of control
2
1
0
***
***
N
V
CVC
GFAP
GAPDH
45 kDa
37 kDa

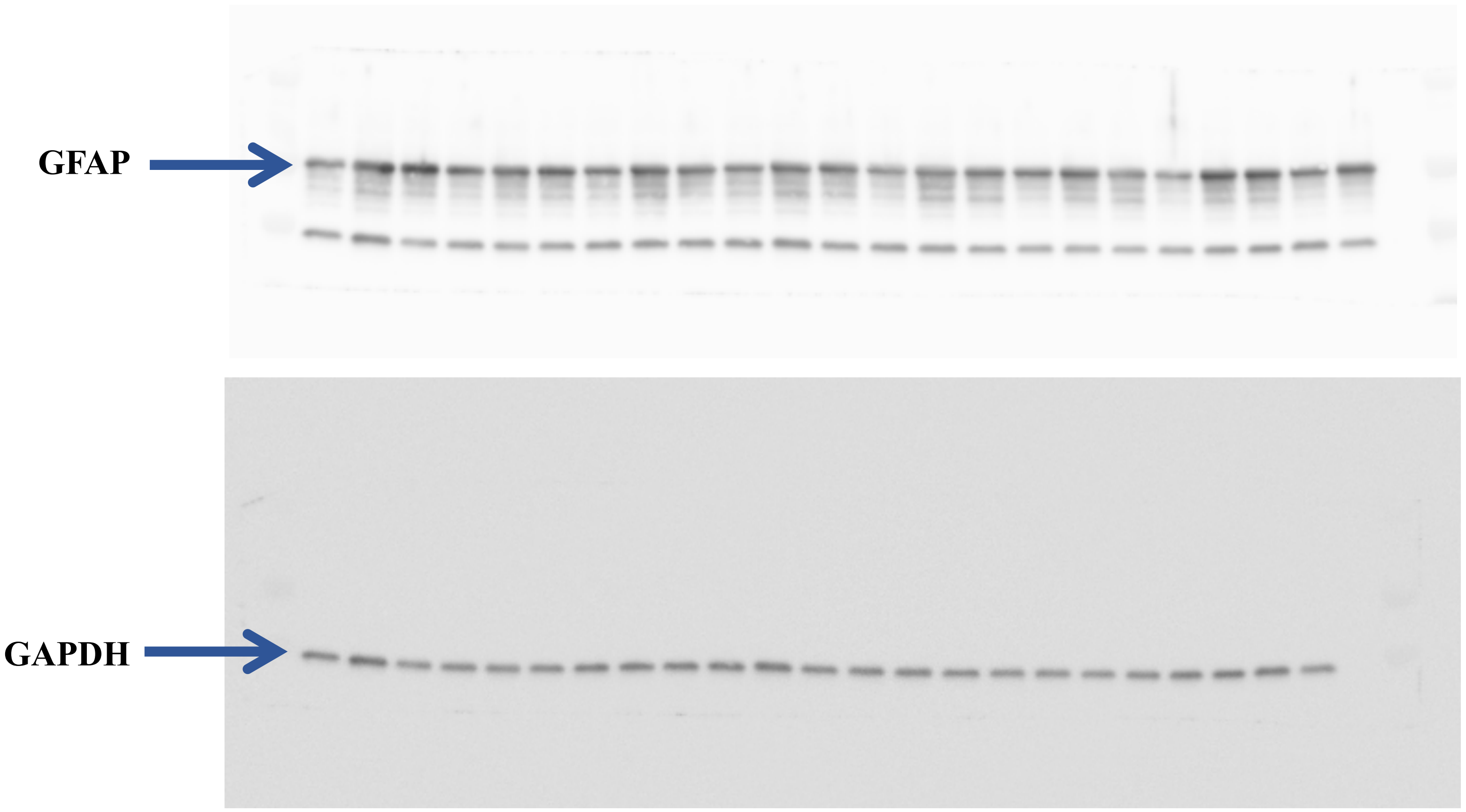

GFAP
GAPDH

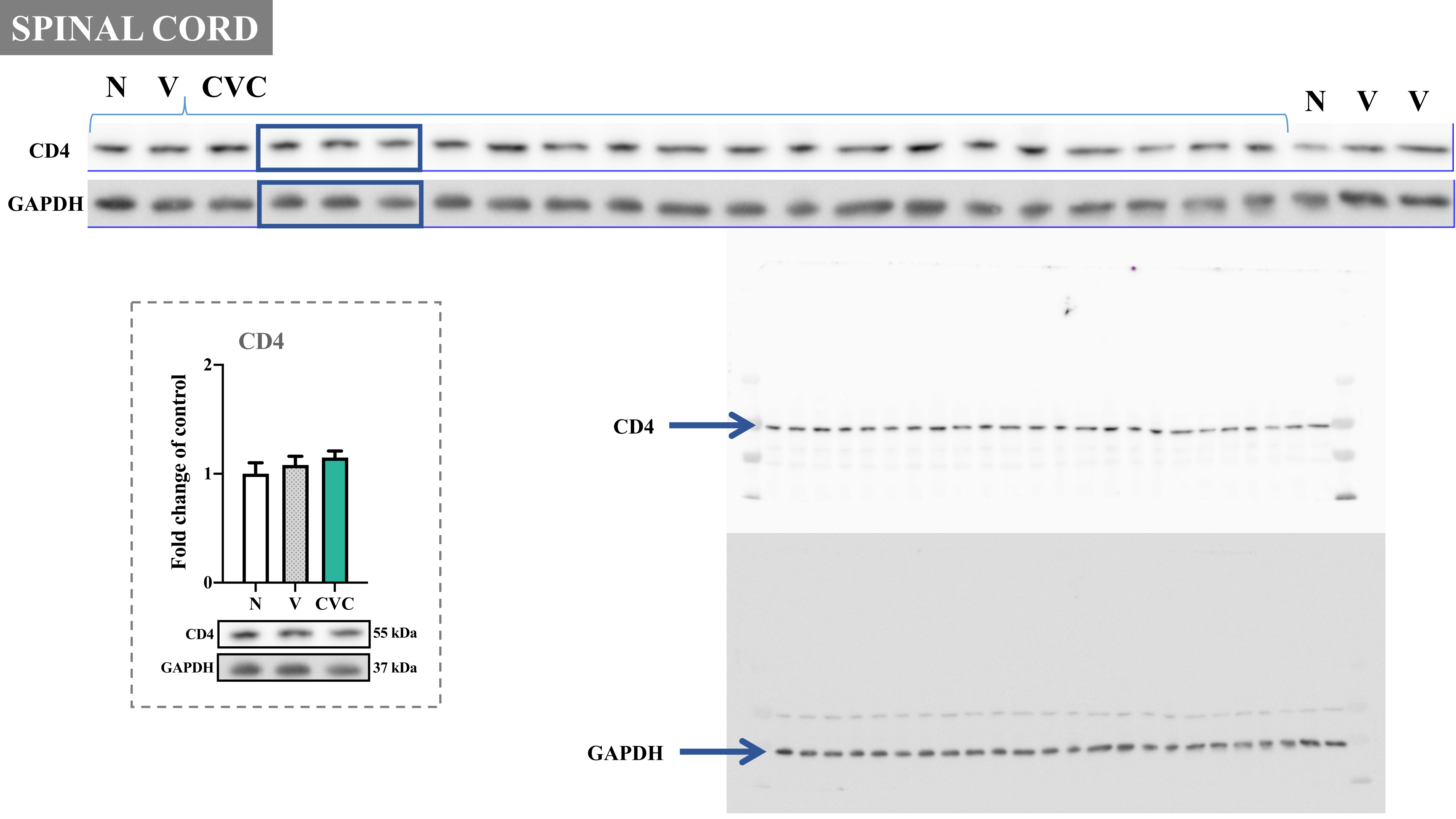

SPINAL CORD
N
V
CVC
N
V
V
CD4
GAPDH
CD4
Fold change of control
2
1
0
N
V
CVC
CD4
55 kDa
GAPDH
37 kDa
CD4
GAPDH

SPINAL CORD

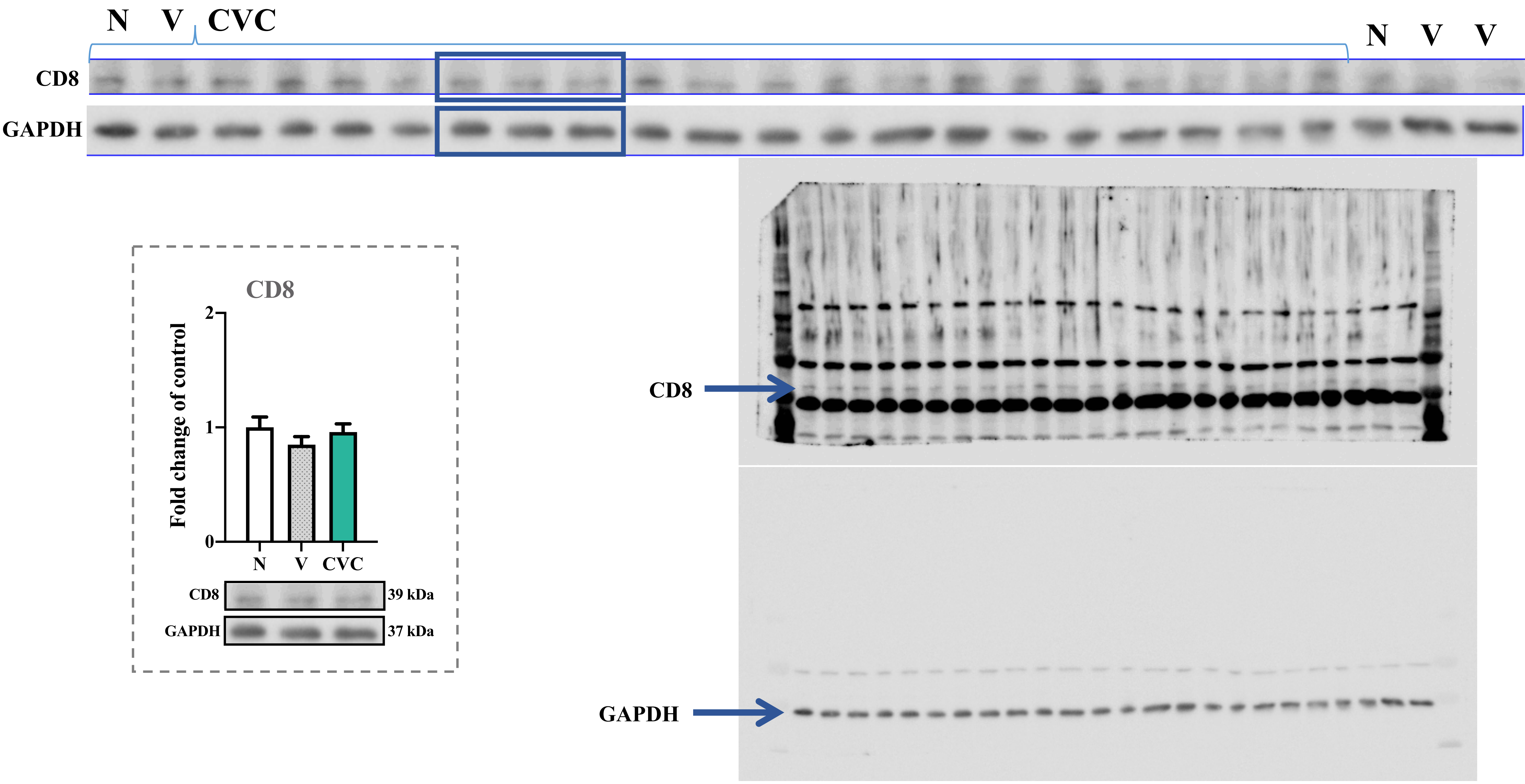
N
V
CVC
N
V
V
CD8
GAPDH
CD8
2
1
0
Fold change of control
N
V
CVC
CD8
39 kDa
GAPDH
37 kDa
CD8
GAPDH

SPINAL CORD

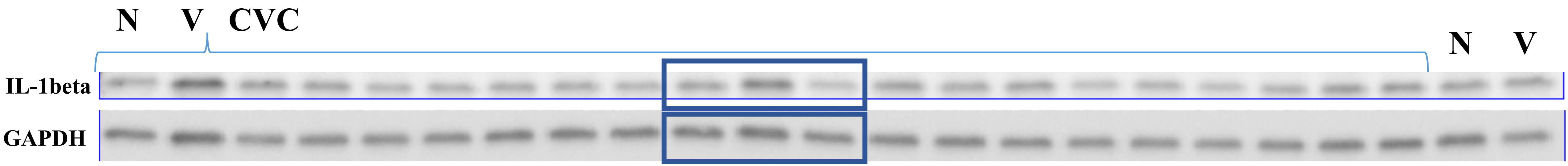

N
V
CVC
N
V
IL-1beta
GAPDH

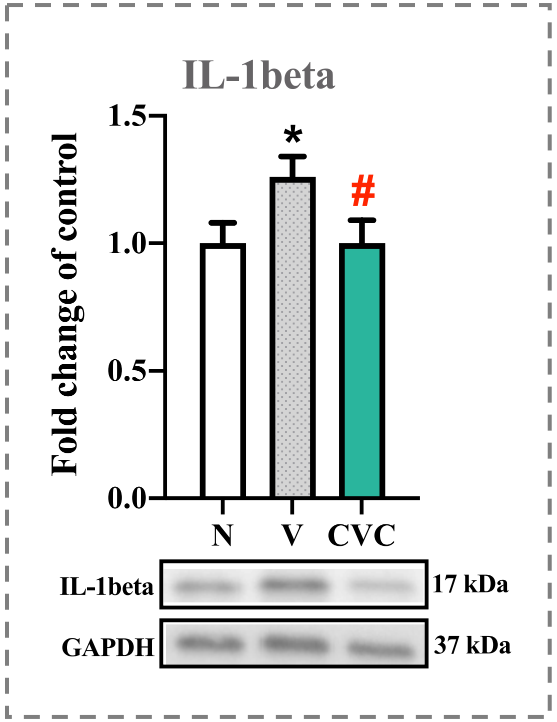

IL-1beta
Fold change of control
1.5
1.0
0.5
0.0
*
#
N
V
CVC
IL-1beta
17 kDa
GAPDH
37 kDa

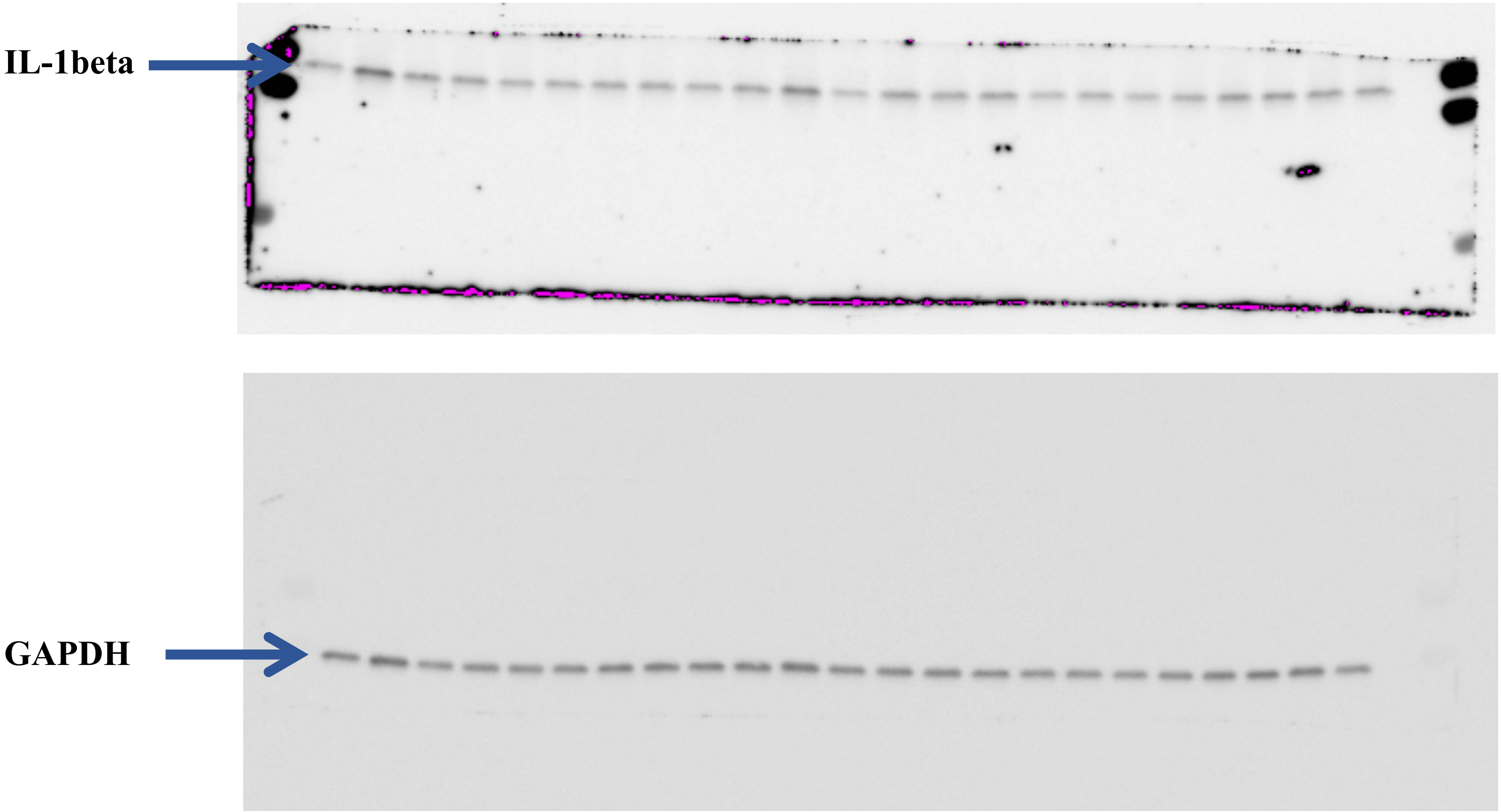

IL-1beta
GAPDH

SPINAL CORD

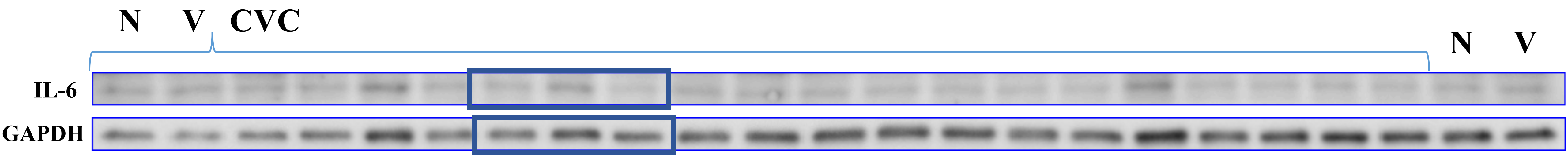

N
V
CVC
N
V
IL-6
GAPDH

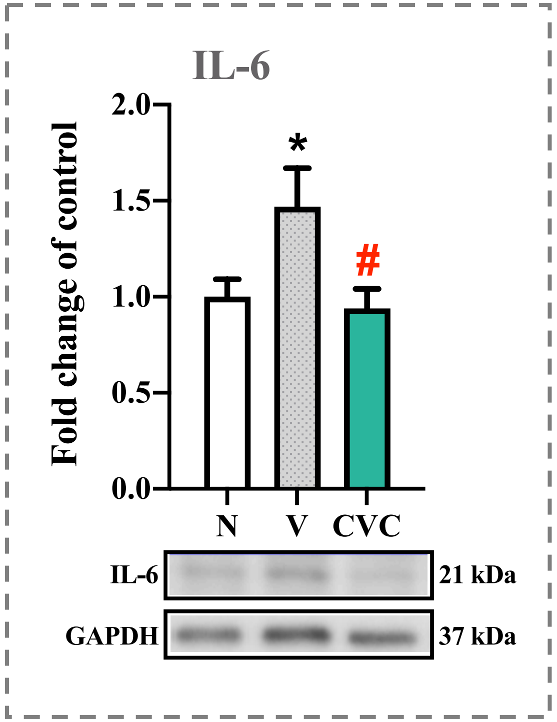

IL-6
2.0
1.5
1.0
0.5
0.0
Fold change of control
*
#
N
V
CVC
IL-6
GAPDH
21 kDa
37 kDa

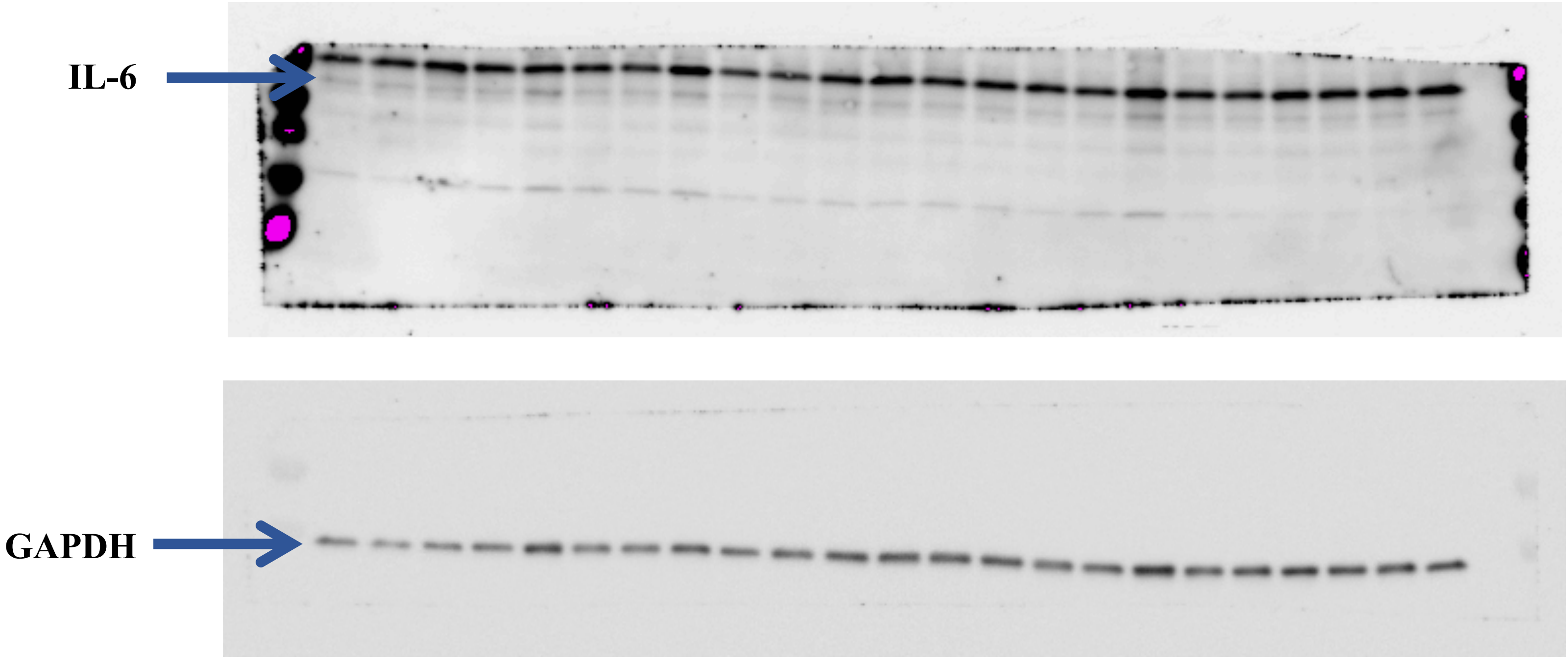

IL-6
GAPDH

SPINAL CORD

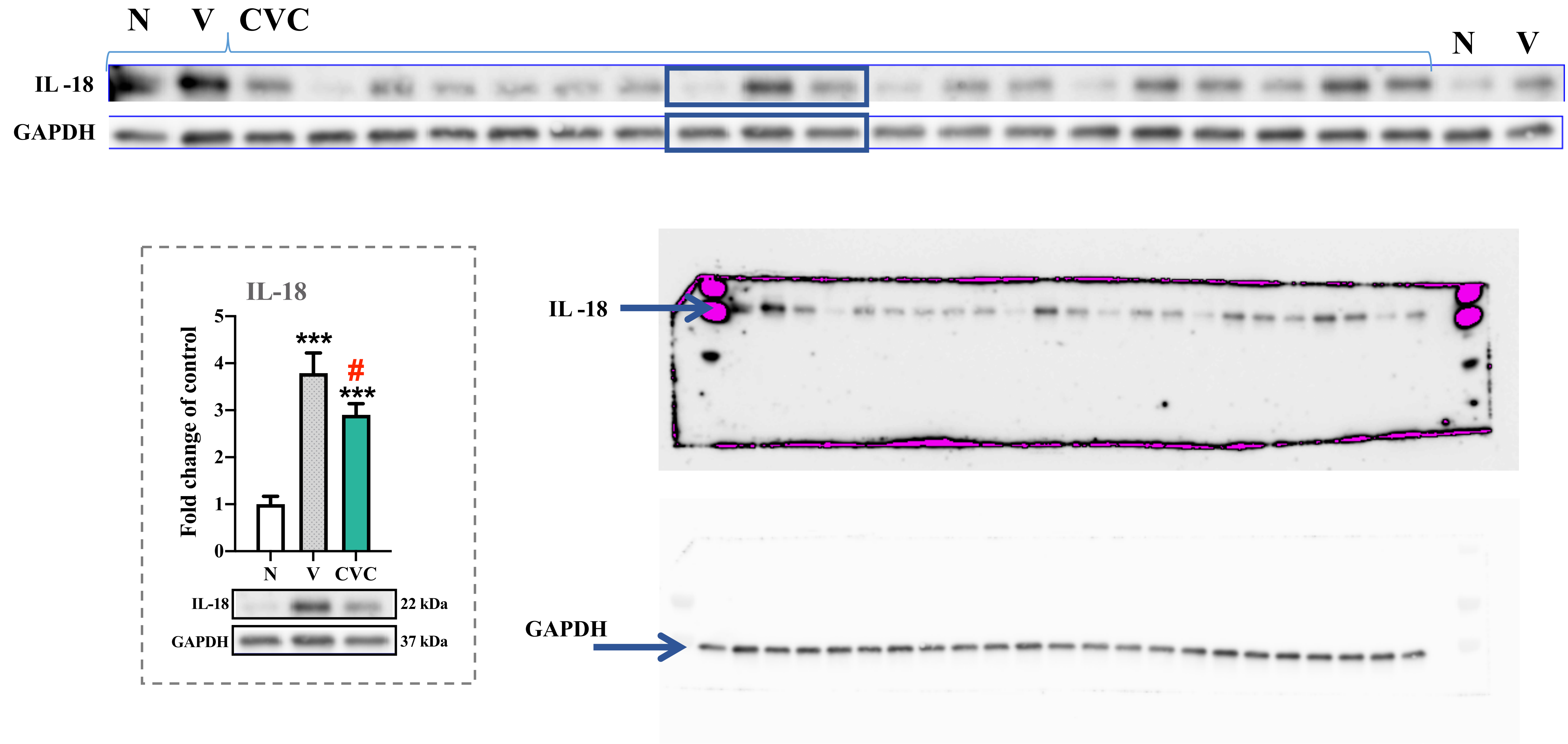
N
V
CVC
N
V
IL -18
GAPDH
IL-18
Fold change of control
5
4
3
2
1
0
***
#
***
N
V
CVC
IL-18
22 kDa
GAPDH
37 kDa
IL -18
GAPDH

DORSAL ROOT GANGLIA

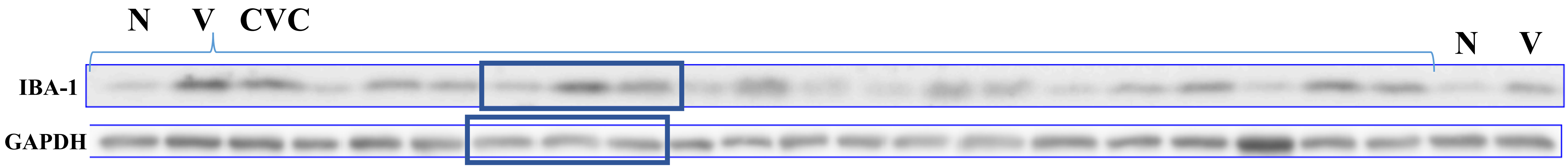

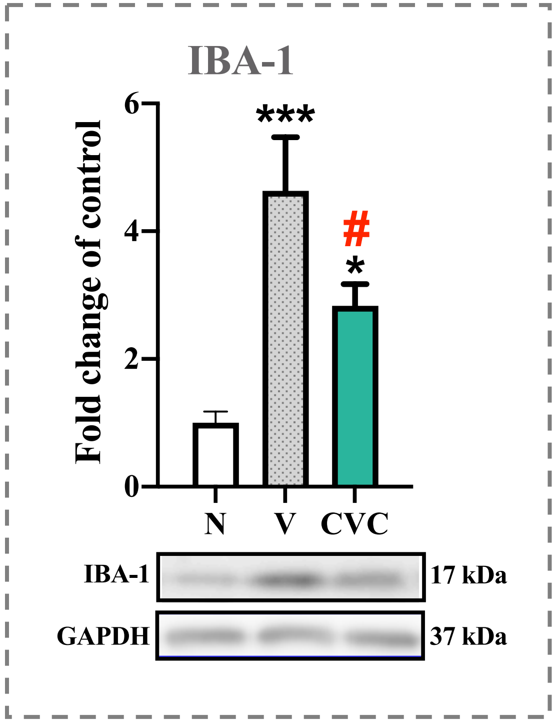

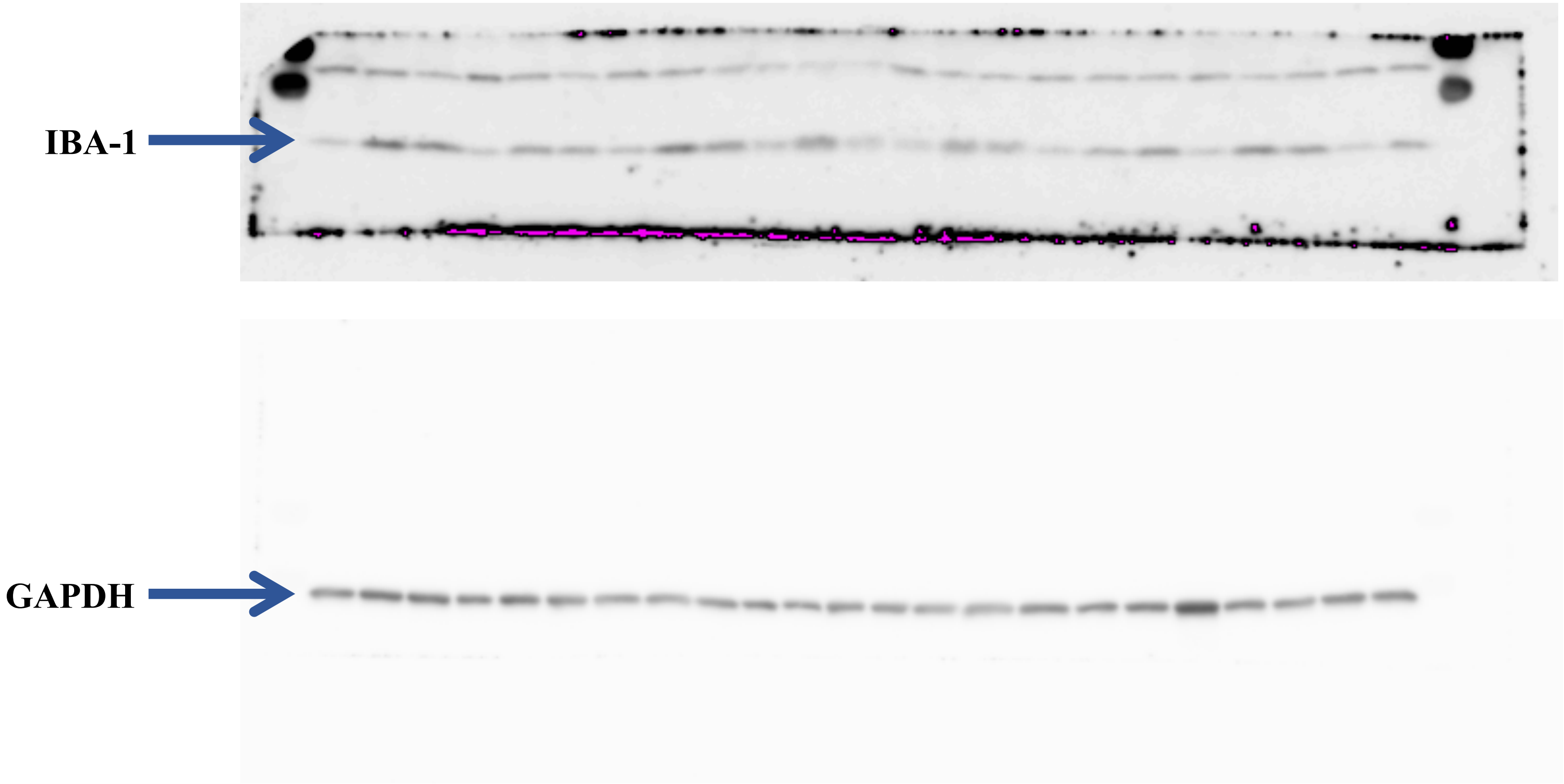

DORSAL ROOT GANGLIA

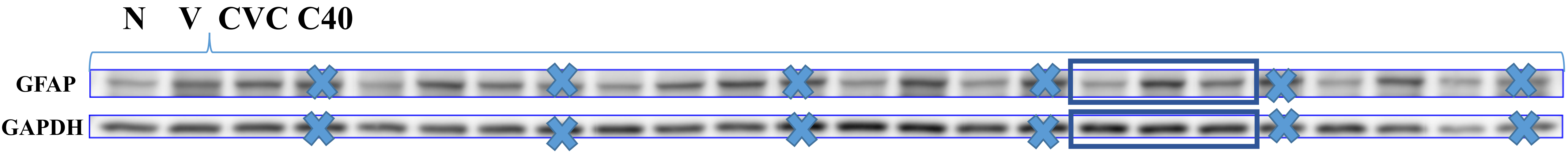

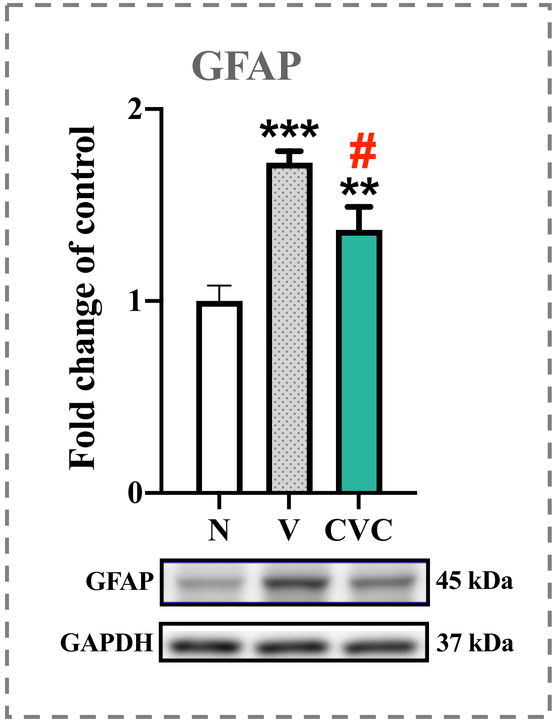

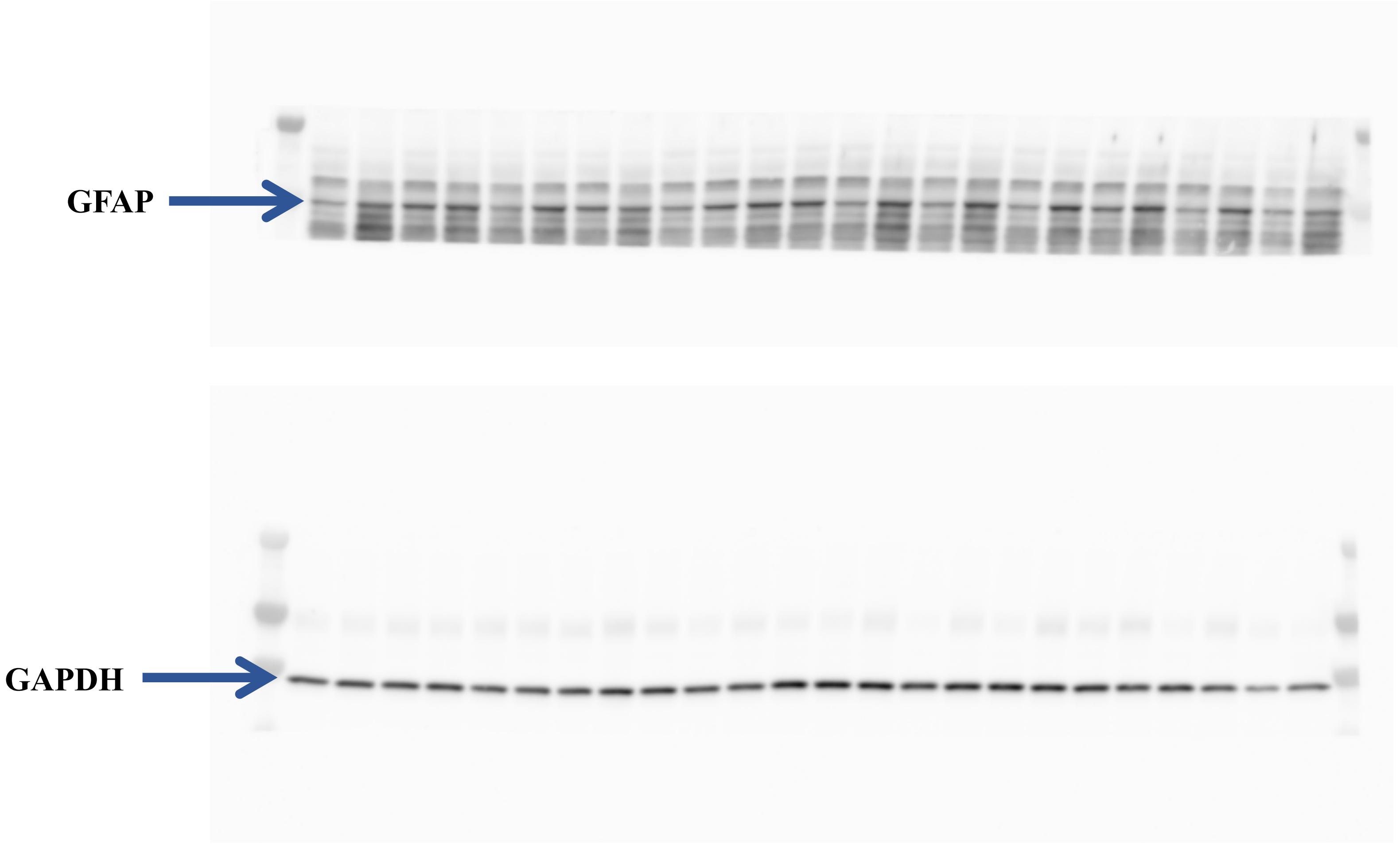

DORSAL ROOT GANGLIA

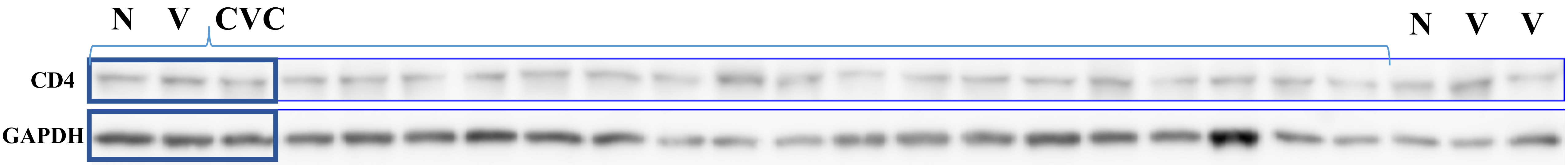

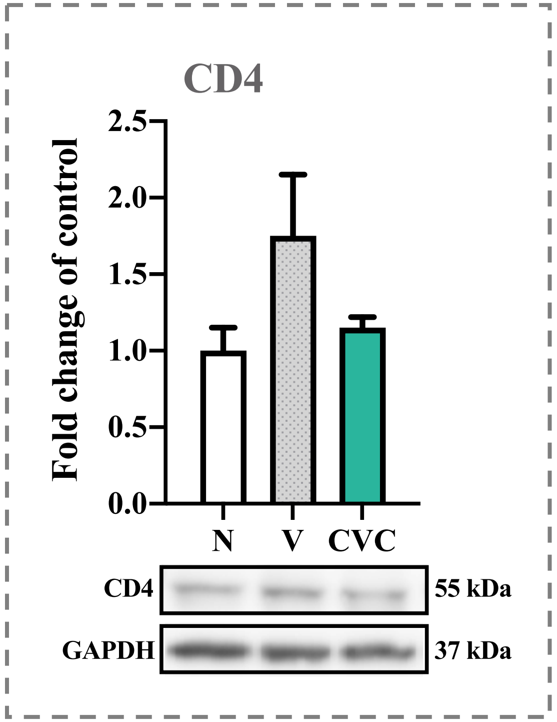

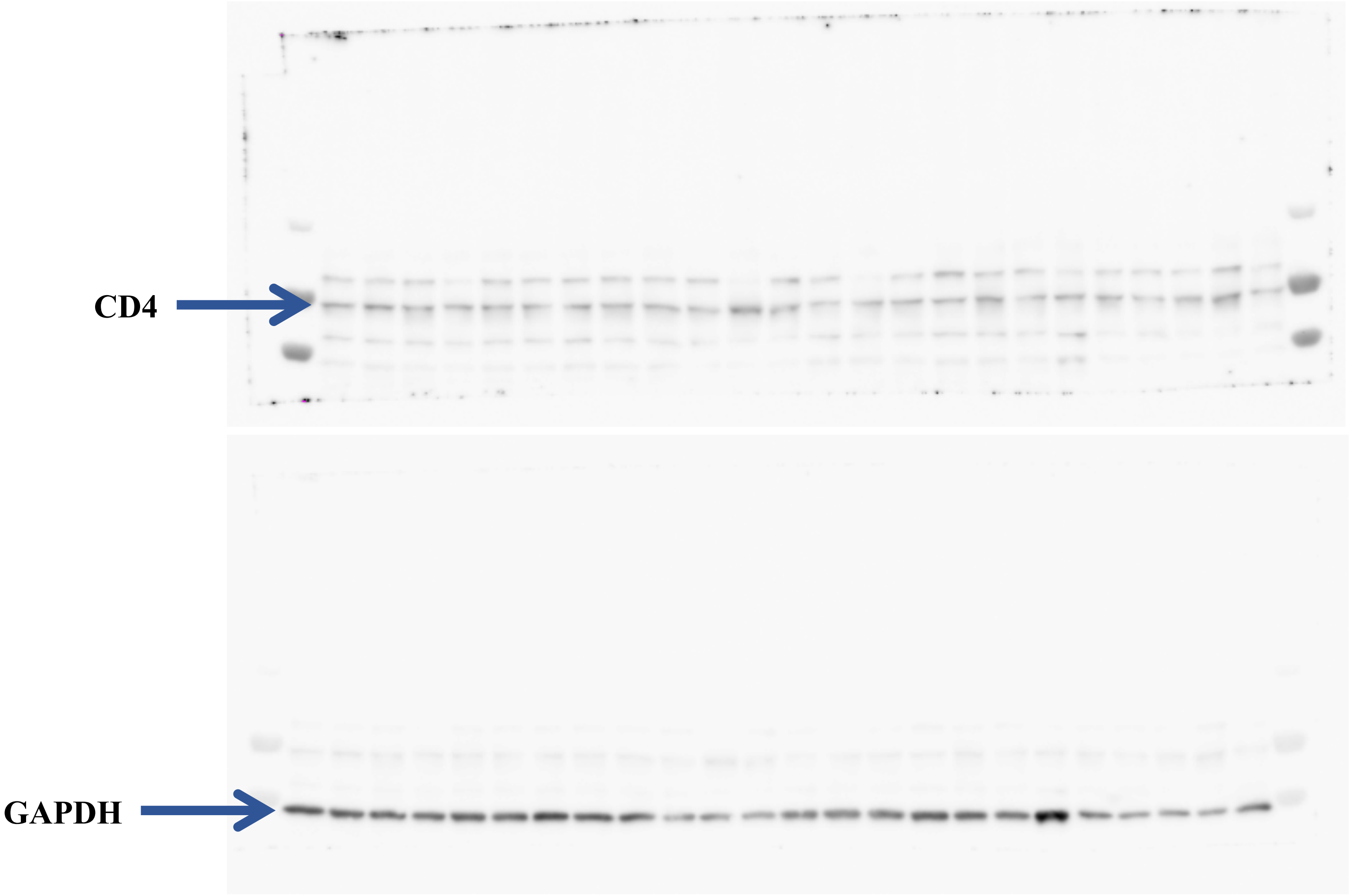

DORSAL ROOT GANGLIA

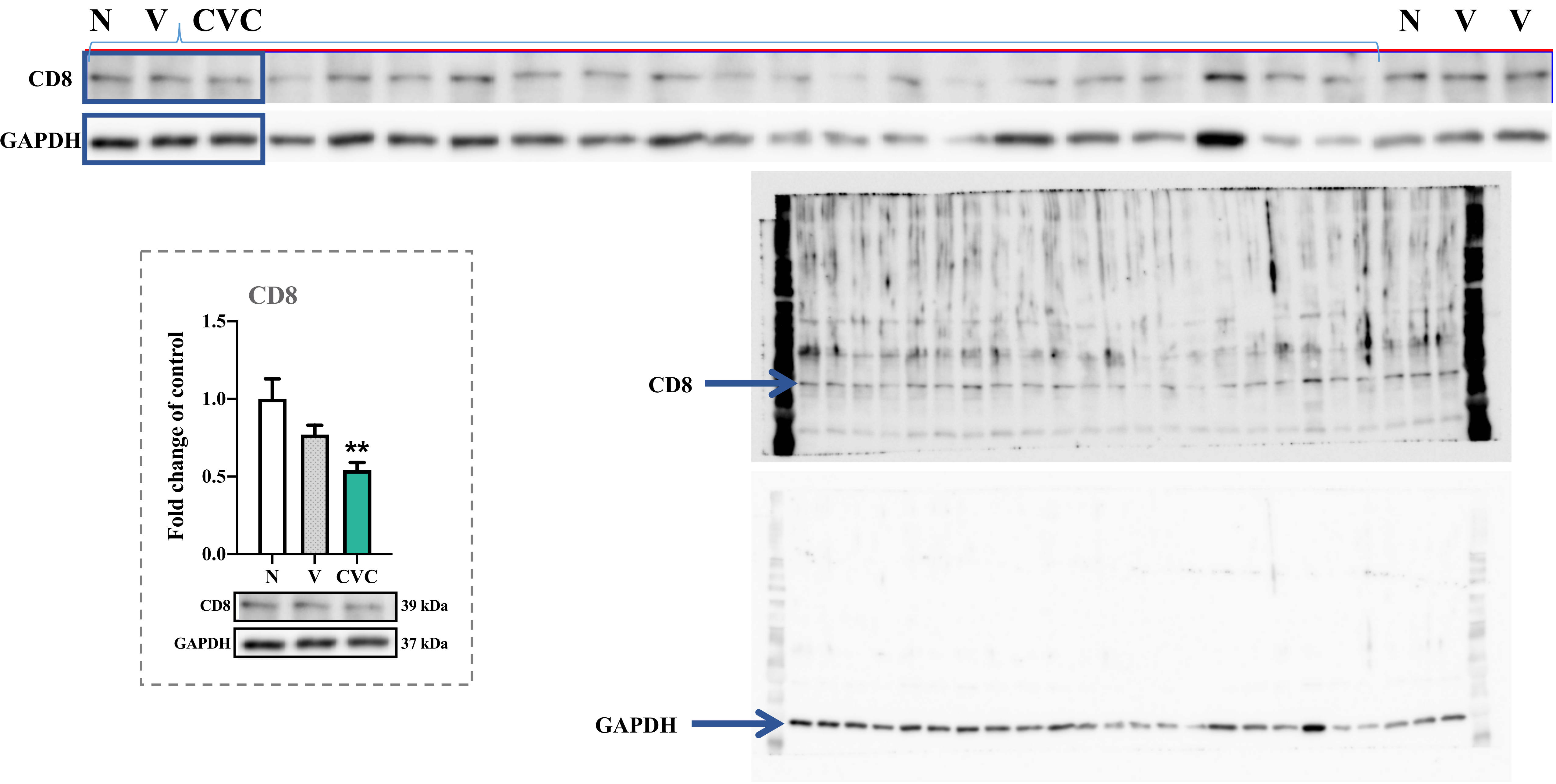

DORSAL ROOT GANGLIA

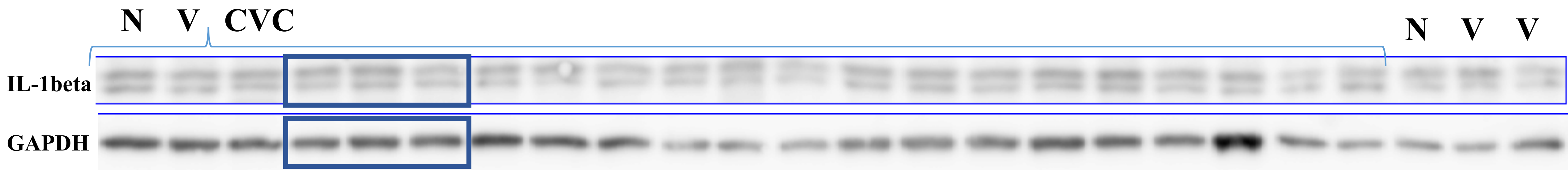

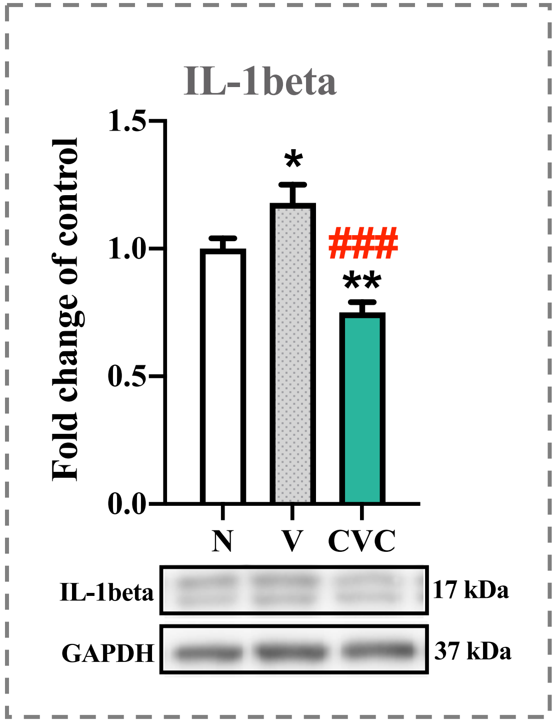

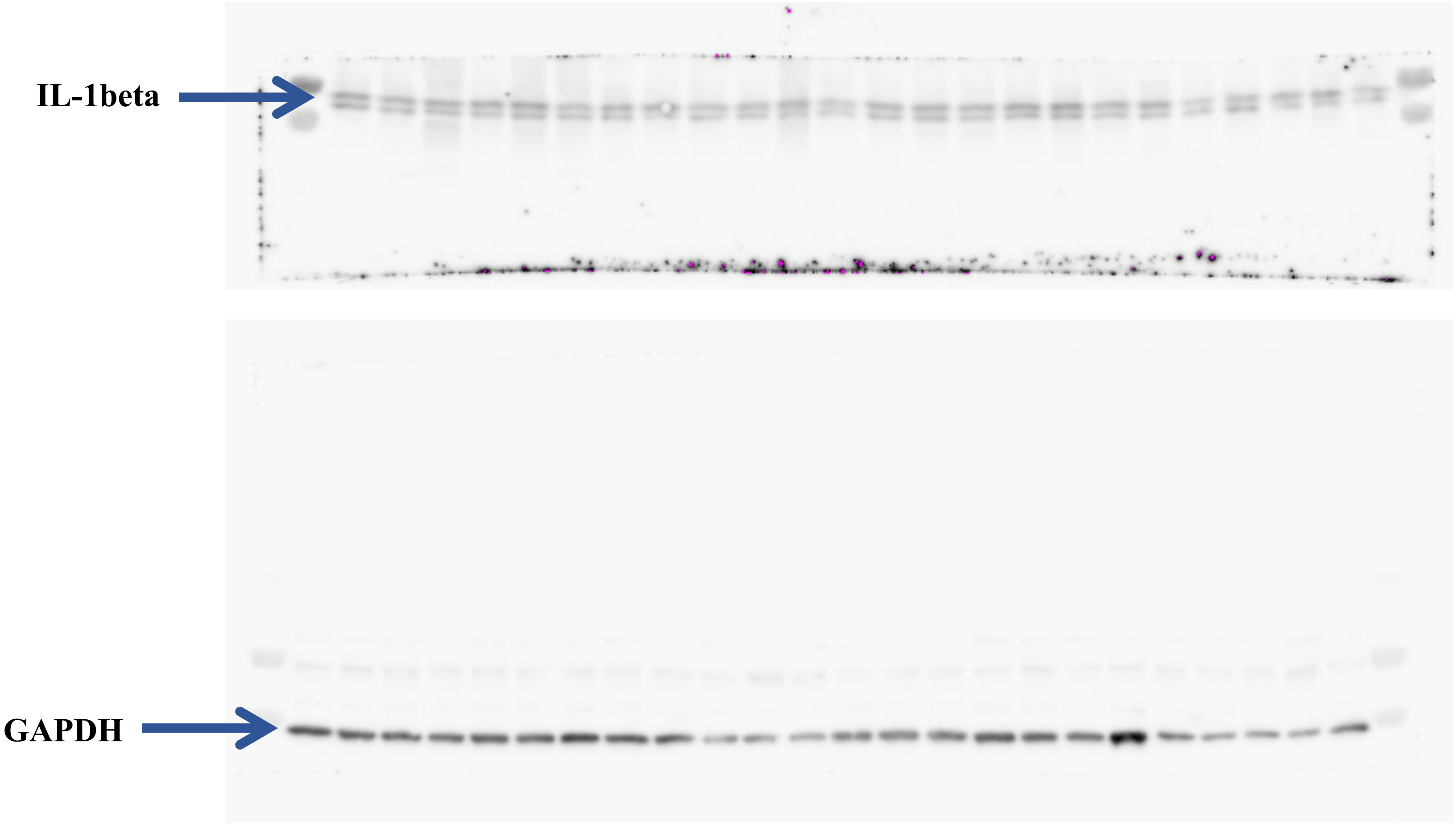

DORSAL ROOT GANGLIA

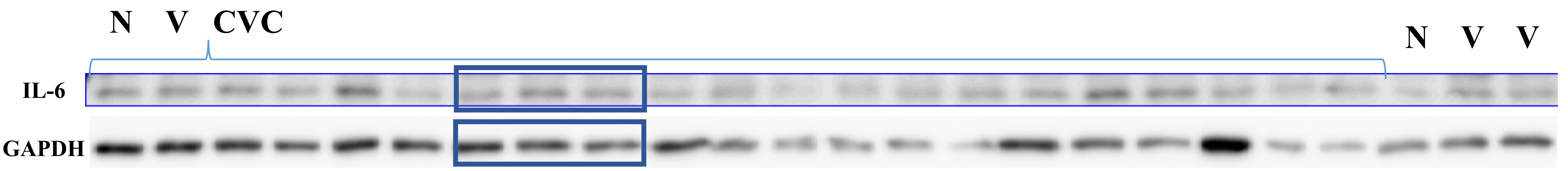

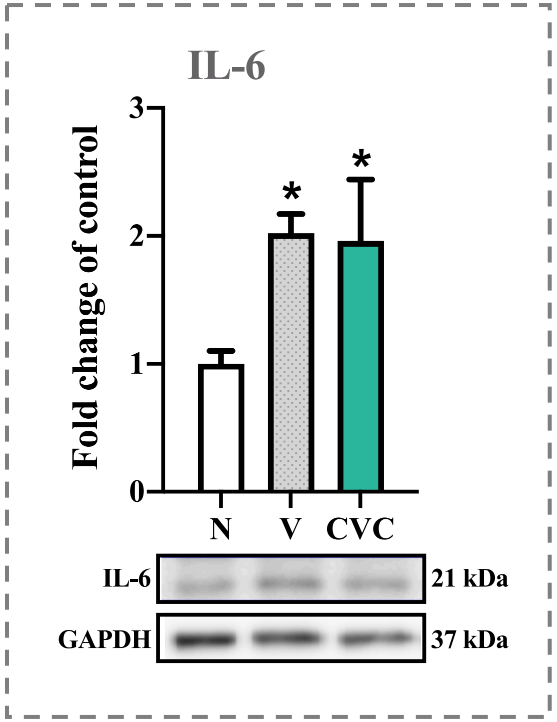

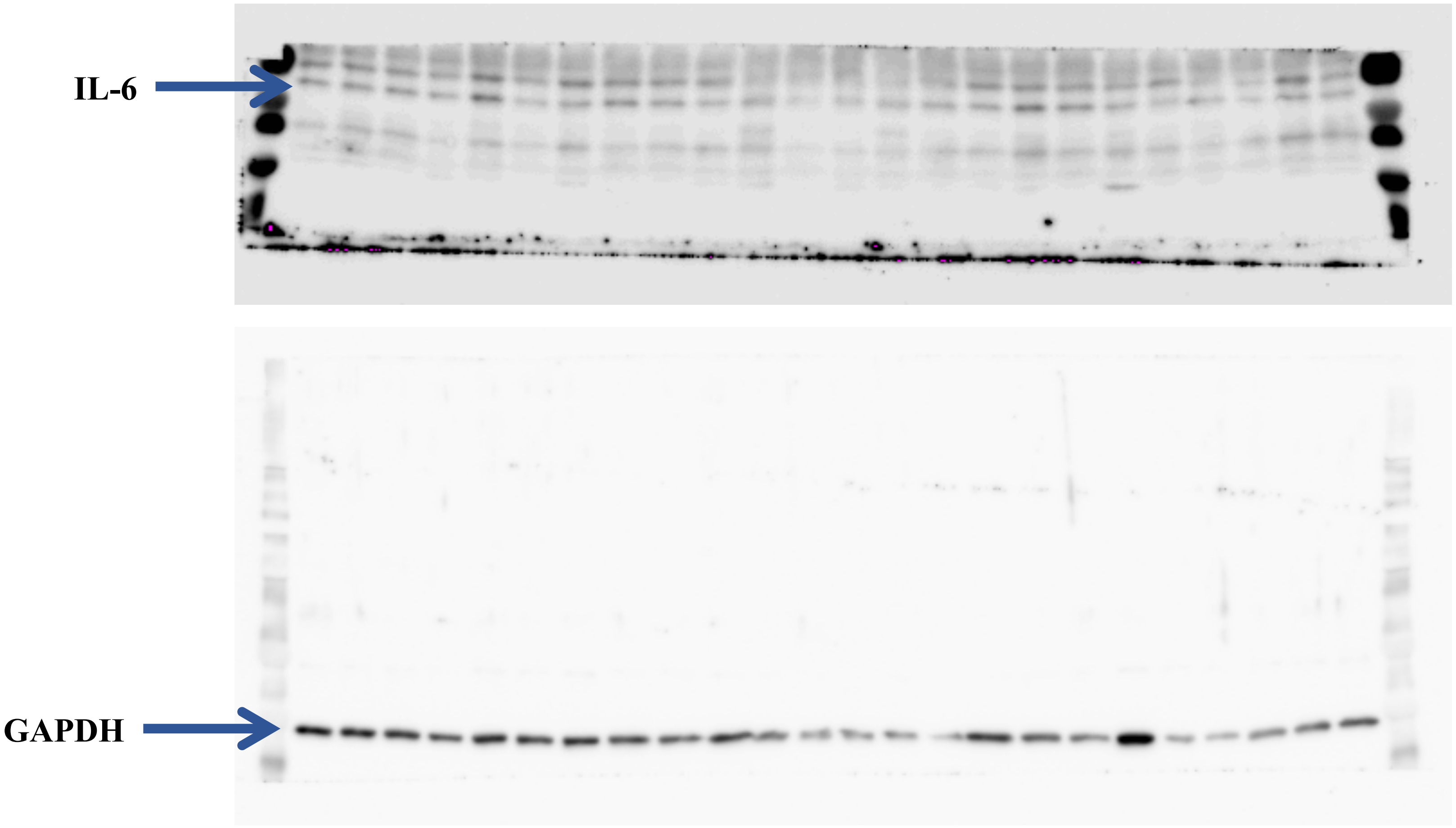

DORSAL ROOT GANGLIA

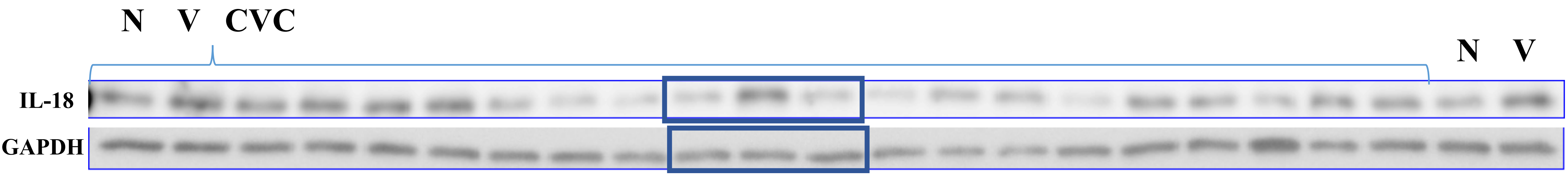

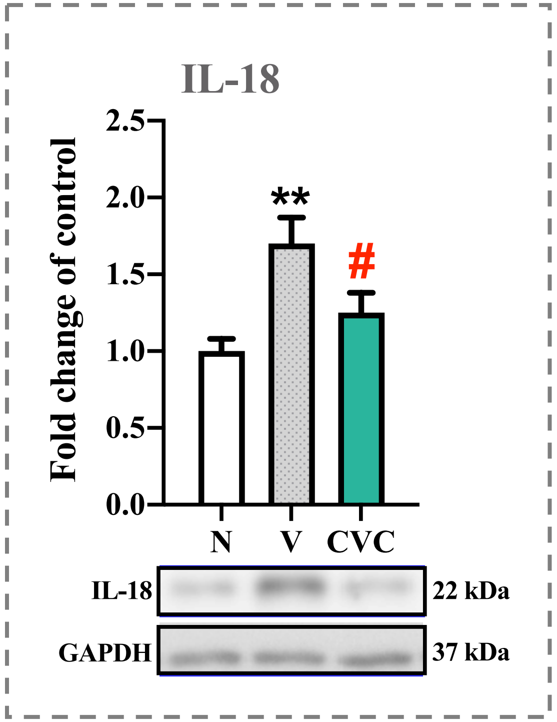

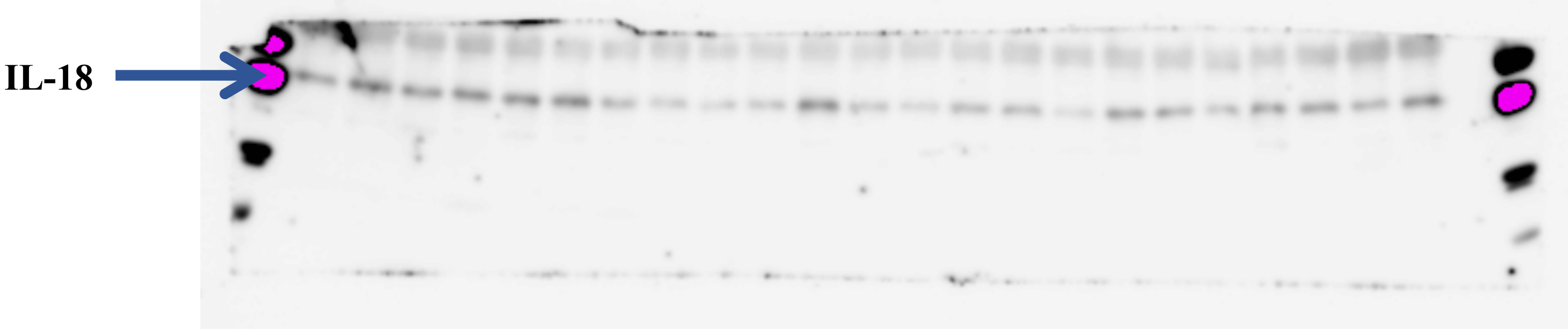

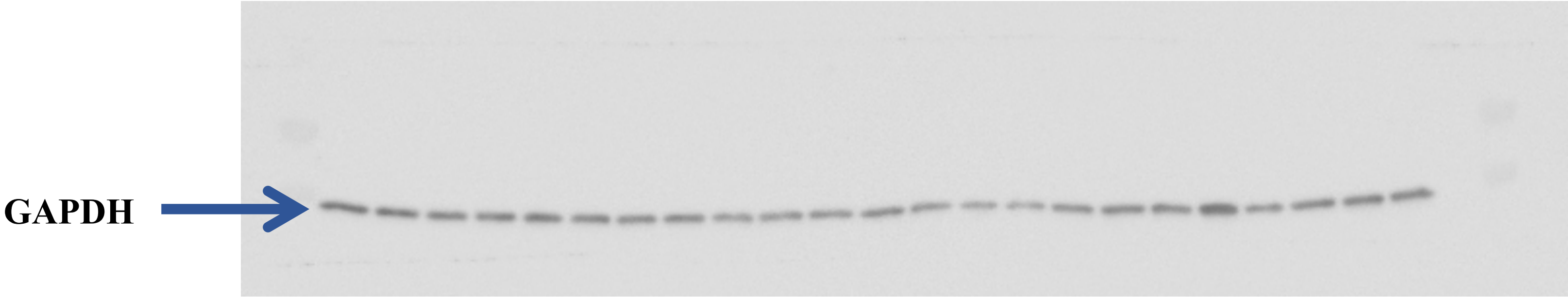

Supplement: Supplementary file 2 [file DataSheet_2.pdf]
